# Multiple *cry* Genes in *Bacillus thuringiensis* Strain BTG Suggest a Broad-Spectrum Insecticidal Activity

**DOI:** 10.3390/ijms241311137

**Published:** 2023-07-06

**Authors:** Alexander Arsov, Maria Gerginova, Tsvetelina Paunova-Krasteva, Kaloyan Petrov, Penka Petrova

**Affiliations:** 1 Institute of Microbiology, Bulgarian Academy of Sciences, 1113 Sofia, Bulgaria; al.arsov@microbio.bas.bg (A.A.); maria_gg@yahoo.com (M.G.); pauny@abv.bg (T.P.-K.); 2 Institute of Chemical Engineering, Bulgarian Academy of Sciences, 1113 Sofia, Bulgaria; kaloian04@yahoo.com

**Keywords:** *Bacillus thuringiensis*, pest control, whole genome sequencing

## Abstract

The properties of *Bacillus thuringiensis* strains as a biopesticide with potent action against moths, beetles, and mosquitoes have been known for decades, with individual subspecies showing specific activity against a particular pest. The aim of the present work is to characterize strains that can be used for broad-spectrum pest control in agriculture. Twenty strains of *B. thuringiensis* were isolated from Bulgarian soil habitats. The strains were screened for genes encoding 12 different crystal (Cry) endotoxins by PCR with specific primer pairs. Seven of the isolates contained *cry* genes in their genomes. *B. thuringiensis* strains PL1, PL3, and PL20 contained at least three different *cry* genes, while *B. thuringiensis* serovar *galleriae* BTG contained at least four. Moreover, scanning electron microscopy (SEM) investigation revealed the production of bipyramidal (PL1, PL3, PL20), polygonal (PL1), cubic (BTG), and spherical crystals (BTG and PL20). Potentially containing the most *cry* genes, the BTG genome was sequenced and annotated. It comprises 6,275,416 base pairs, does not contain plasmids, has a GC content of 35.05%, and contained 7 genes encoding crystal toxins: *cry1Ab35*, *cry1Db*, *cry1Fb*, *cry1Ib*, *cry2Ab*, *cry8Ea1*, and *cry9Ba*. This unique combination would possibly enable the simultaneous pesticidal action against pest species from orders Lepidoptera, Coleoptera, Diptera, and Hemiptera, as well as class Gastropoda. Whole-genome sequencing provided accurate information about the presence, localization, and classification of Cry toxins in *B. thuringiensis* BTG, revealing the great potential of the strain for the development of new broad-spectrum bio-insecticides.

## 1. Introduction

According to the Food and Agriculture Organization of the United Nations (FAO), the world’s agriculture loses between 20% and 40% of its total production due to pest attacks every year. Invasive insects alone cause over USD 70 billion in losses to the global economy [1]. More than 10,000 pest species attack cultivated plants, feed on their above-ground and underground parts, and fatally destroy the crops. Most plant damage is caused by insects and their larvae (moths, whiteflies, beetles, and thrips), nematodes, and slugs. *Bacillus thuringiensis*, a common soil-dwelling bacterium, has been known for decades as a good option for an environmentally friendly, safe for humans, species-specific, and highly effective pest biocontrol agent [2,3,4,5].

*B. thuringiensis* was isolated in the early 20th century, first in Japan as a silkworm pathogen, and later by Berliner from the Mediterranean flour moth *Anagasta kuehniella* in the Thuringia province of Germany [6]. Scientists and farmers quickly saw the potential of the new bacterial species for pest control, and the earliest commercial product to contain it was launched in France in 1938 under the name Sporeine. Increased interest in *B. thuringiensis* is attributed to Steinhaus, who obtained pure cultures in 1942 and drew attention to their insecticidal potential, followed by the discovery of Angus, who demonstrated that protein crystals formed during sporulation were responsible for the insecticidal action [7].

Toxigenic strains of the species *B. thuringiensis* contain genes that encode δ-endotoxins, also known as Cry, or crystalline, due to their ability to auto-crystallize in vivo. When ingested by the insect or its larvae, the crystal protoxins are activated by proteases in the digestive system. The active toxins bind to specific receptors on the cells of the midgut, disrupt the membrane transport, and cause the death of the insect or its larvae after 2 to 48 h [8,9,10,11,12]. Currently, two molecular models describe Cry toxins’ insecticidal mechanisms of action: the sequential binding model, which suggests the formation of cell membrane pores, and the signaling pathway model describing a signaling cascade of cell apoptosis induction [13].

*B. thuringiensis* is a Gram-positive, catalase-positive, oxidase-negative, strictly aerobic bacterium with peritrichous flagella that allow motility. It develops in two life cycles: vegetative growth and sporulation. Cry protein crystals are formed during the sporulation phase [14,15,16,17], which involves seven distinct stages: (1) axial filament formation; (2) formation of the pre-spore septum; (3) parasporin crystals and pre-spore formation; (4–6) formation of exospores, development of the primary cell wall and transformation of spore nucleoids; (7) spore maturation and cell lysis [8]. Crystals are synthesized after the second stage of sporulation and accumulate in the cell, where they can represent up to 30% of the dry weight of sporulated cells [18]. The shapes of the parasporal crystals can be bipyramidal, pyramidal, cubic, flat rhombic, spherical, or rectangular [19,20,21,22] and may include one or more endotoxins, with a molecular weight between 60 kDa and 140 kDa [13].

More than 700 different *cry* genes have been sequenced and described so far [23]. According to their amino acid sequence, Cry toxins have been classified into 16 groups with 272 holotypes [2]. They usually consist of three reserved domains, each exhibiting a specific function [13]. Domain I (pfam03945) is located at the N-terminus of the protein; it consists of seven alpha helices and is involved in pore formation. The middle domain, Domain II (pfam00555), has a beta-prism structure and is responsible for the interaction with mid-intestinal epithelial cell membrane receptors of target insects determining the pesticidal specificity [24]. Domain III (cd04085), the C-terminus domain, forms a regular β-sandwich structure composed of two antiparallel β-sheets and is critical for receptor interaction and structural stability. Domain III is usually involved in the specific binding to receptors such as N-acetylgalactosamine [25].

The different classes of *B. thuringiensis* Cry toxins bind to various receptors (cadherins, aminopeptidases, alkaline peptidases, ATP-binding transporter proteins) and therefore bioinsecticide preparations containing *B. thuringiensis* strains have specific toxicity against certain pest groups [23,26]. Thus, the bioinsecticide formula based on *B. thuringiensis* ssp. *kurstaki* (Sumitomo Chemical Co., Ltd., Tokyo, Japan—Valent Biosciences’s DiPel^®^) is directed towards lepidopteran larvae only, the insecticide containing *B. thuringiensis* ssp. *galleriae* (Green Earth Ag & Turf LLC, Branford, CT, USA) is used solely against scarab beetles, while *B. thuringiensis* ssp. *israelensis* (Arbico Organics LLC, Oro Valley, AZ, USA) controls only Diptera representatives (mosquitoes and black flies). In addition to these limitations, due to the long-term use of particular insecticidal strains, an inherited resistance to certain formulae, such as DiPel^®^, has been developed by a number of pest species [27,28]. Today, genetic engineering of *B. thuringiensis* is increasingly used to obtain strains with a broader spectrum and stronger pesticidal activity [29,30,31].

The aim of the present work is to propose an alternative solution to the problems described herein by searching for *B. thuringiensis* strains containing new *cry* genes or unique combinations of them, which would enable a more efficient fight against pests from different taxonomic groups. Based on the available knowledge about the toxicity and specificity of Cry proteins, studying the genetic profile of newly isolated strains can be used to predict their potential for pest control.

## 2. Results

### 2.1. Isolation and Identification of New B. thuringiensis Strains

To select new crystal toxin producers, we collected *B. thuringiensis* isolates from remote agricultural and mountainous regions of Bulgaria. Twenty strains of *B. thuringiensis* were isolated from biopesticide-untreated soil samples taken from rice, sunflower or maize fields, wastelands, and forests of the Rhodope Mountains.

The initial identification of the strains was based on cells and colony morphology (rod-shaped cells, creamy white to light beige colonies with ragged edges).

**Figure 1:**
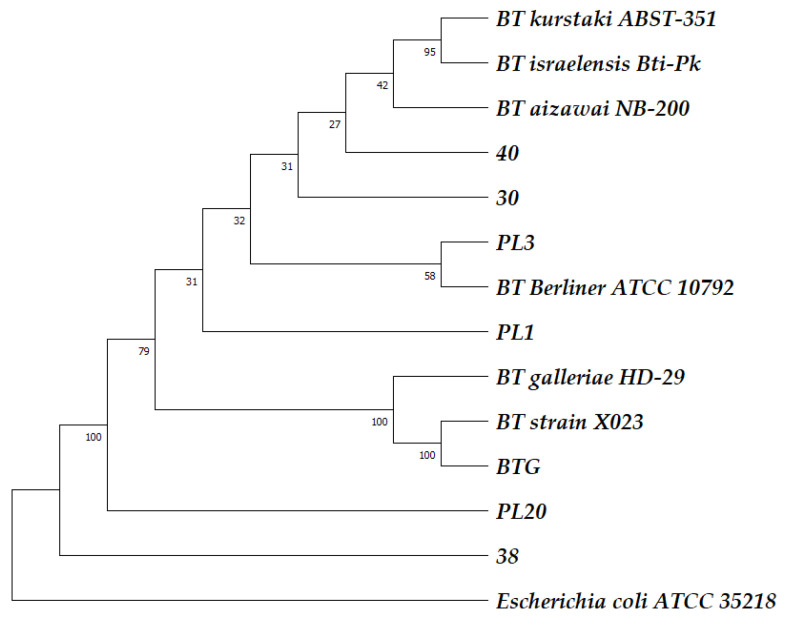
Bootstrapped phylogenetic tree of the newly isolated strains PL1, PL3, PL20, BTG, 30, 38, and 40 based on 16S rRNA gene sequences inferred using the UPGMA method [32]. The evolutionary distances were computed using the Maximum Composite Likelihood method [33]; evolutionary analyses were conducted in the MEGA11 program [34]. In the comparison sequences, the following NCBI GenBank accession numbers were used: OR084785 (PL1), OR084784 (PL3), OR084783 (PL20), JASNQP000000000 (BTG), OR084786 (30), OR084787 (38), OR084788 (40), MN396730 (ATCC 10792), NZ_CP083101 (ABTS-351), OL721864 (Bti-Pk), CP010089 (HD29), GCA021651035 (X023), and AM980865.1 (ATCC 35218).

Determination of isolates to species level was done by 16S rRNA gene sequencing and BLAST comparison of the obtained sequences with the NCBI GenBank database. In parallel, the total DNA of the strains was tested for the presence of *cry* genes. Figure 1 presents the phylogenetic positions of the isolates, which is affiliated with *B. thuringiensis* species and were positive for *cry* genes (PL1, PL3, PL20, BTG, 30, 38, and 40).

Nucleotide sequence similarity of the 16S rRNA genes showed that the isolates PL1, 30, and 40 were clustered with the branch *B. thuringiensis* subsp. *kurstaki*/*israelensis*/*aizawai*. This identifies the strains as belonging to the *B. thuringensis* species but does not provide information on the subspecies as due to the high similarity in 16S rDNA these three subspecies cannot be distinguished by this method. The strain PL3 was close to *B. thuringiensis* subsp. *Berliner*, while the strain BTG was clustered with *B. thuringiensis* subsp. *galleriae*, and *B. thuringiensis* X023 (*Bt*X023), a strain with high insecticidal activity isolated in Hunan Province, China [19]. Strains 38 and PL20 fell into separate clusters. Accurate subspecies assignment based on whole genome sequencing was done only for strain BTG.

### 2.2. PCR Screening for Genes Encoding Cry δ-Toxins in Newly Isolated B. thuringiensis Strains

The methodology used to study *B. thuringiensis* strains and their potential for toxin synthesis included primer design, isolation, and purification of genomic DNA from the tested strains, PCR, sequencing, and bioinformatic analysis of the obtained fragments. Aiming to detect a broad Cry toxins spectrum, the presence of 12 toxins was tested: *cry1Aa*, *cry1Ab*, *cry2Aa*, *cry3Aa*, *cry5A*, *cry7Aa*, *cry8Ba2*, *cry9Ca*, *cry9Da*, *cry11A*, *cry15A*, and *cry22A* (Table 1).

*B. thuringiensis* serovar *kurstaki* ABTS-351 (the strain in the composition of the commercial preparation DiPel^®^) was used as a positive control for genes 1Aa, 1Ab, and 2Aa. Seven of the newly isolated *B. thuringiensis* strains possessed genes encoding different toxins. PCR products analysis showed that PL1, PL3, and PL20 contained at least three *cry* genes each, while *B. thuringiensis* strain BTG contained at least four. The other collected strains did not yield PCR products for *cry* genes and were not analyzed further. The majority of the fragments obtained were unique and amenable to sequencing. The study of homology of *cry1Ab* partial sequences of *B. thuringiensis* strains PL1, PL3, and PL20 showed that they are 100% homologous to *cry1Ab* of *B. thuringiensis* strain ABTS-351 (MK184462, 3468 bp). However, the primers targeting *cry7Aa* and *cry8Ba2* did not generate a single PCR product, hence, the existence of these toxin genes had to be confirmed by another method.

### 2.3. Observation of B. thuringiensis Spores and Crystal Toxins by Scanning Electron Microscopy (SEM)

**Figure 2:**
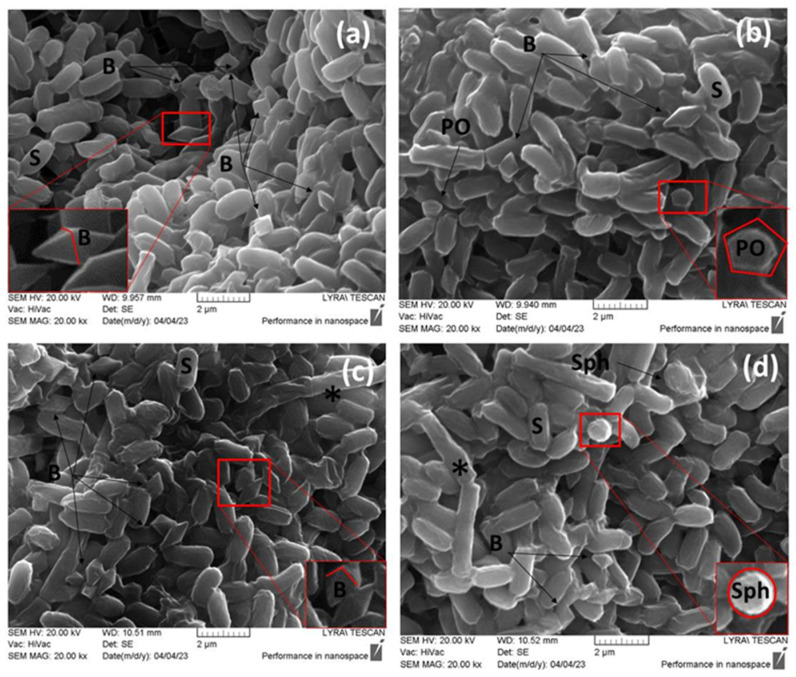
Scanning electron microscope (SEM) images demonstrating the synthesis of crystalline toxins by *B. thuringiensis* strains PL1 and PL20. SEM micrographs show the surface architecture of three different types of synthesized crystals, marked with black arrows. (**a**) *B. thuringiensis* PL1, bipyramidal crystals; (**b**) *B. thuringiensis* PL1, polygonal crystals; (**c**) *B. thuringiensis* PL20, bipyramidal crystals; (**d**) *B. thuringiensis* PL20, spherical crystals. Designations: B, bipyramidal crystal; PO, polygonal crystal; Sph, spherical crystal; S, spore; black star—vegetative cells. The higher magnification shows the geometry of the crystal. Bar = 2 µm.

**Figure 3:**
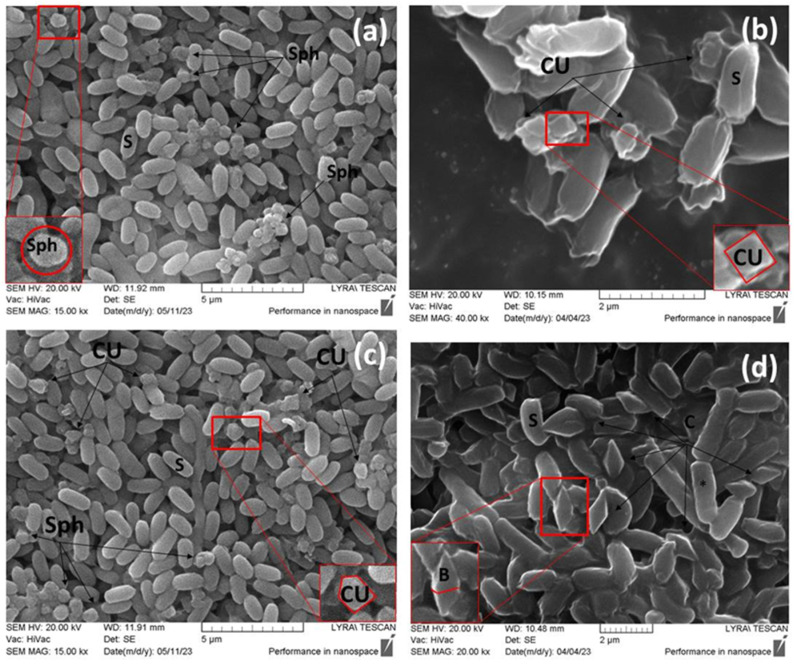
Scanning electron microscope (SEM) images demonstrating the synthesis of crystalline toxins by *B. thuringiensis* strains BTG and PL3. SEM micrographs show the surface architecture of three different types of synthesized crystals, marked with black arrows. (**a**) *B. thuringiensis* BTG, spherical crystals; (**b**) *B. thuringiensis* BTG, cubic crystals; (**c**) *B. thuringiensis* BTG, cubic crystals; (**d**) *B. thuringiensis* PL3, bipyramidal crystals. Designations: B, bipyramidal crystal; CU, cubic crystal; Sph, spherical crystal; S, spore; black star—vegetative cells. The higher magnification shows the geometry of the crystal; Bar = 2–5 µm.

The four strains, namely PL1, PL3, PL20, and BTG, were designated as promising due to their possession of multiple genes encoding Cry proteins. The strains were characterized by their ability to form spores and crystal toxins. The crystal morphology observed by scanning electron microscopy (SEM) is shown in Figure 2 and Figure 3.

All images showed the presence of spores and parasporal crystal forms, attached or not attached to the spores. Three of the strains were capable of releasing two different morphological types of crystals. *B. thuringiensis* strain PL1 produced bipyramidal and polygonal crystals (Figure 2a,c), whereas PL20 formed bipyramidal and spherical crystals (Figure 2c,d).

The most impressive was the crystal composition of *B. thuringiensis* strain BTG, which released spherical (Figure 3a,c) and cubic crystalline forms (Figure 3a,b). Unlike the others, *B. thuringiensis* PL3 formed only one type of crystal inclusion, which was bipyramidal in shape. However, despite the similar shape, the bipyramidal crystals formed by *B. thuringiensis* PL3 were significantly larger in size compared to those of PL1 and PL20 (Figure 3d).

### 2.4. Whole Genome Sequencing (WGS) of B. thuringiensis BTG

*B. thuringiensis* BTG carried at least four different *cry* genes in its genome and released two different types of crystal toxins. Being the most promising, it was selected for whole genome sequencing (WGS), since the discovery of more genes encoding crystal proteins in its genome was expected.

The circular chromosome of *B. thuringiensis* BTG consists of 6,275,416 base pairs, with a G + C content of 35.05%. The BTG genome was de novo assembled in 153 contigs, bearing 6026 genes (5909 complete CDS), 102 genes for tRNAs, 14 for rRNAs, and 1 encoding transfer-messenger RNA (tmRNA). The largest part of them is engaged in amino acid and carbohydrate transport and metabolism (581 genes) and in energy production and conversion (195 genes). A comparison of the BTG genome with the NCBI database shows 94.77% homology with the genomes of bacteria of the genus *Bacillus* and 5.23% homology with the genus *Pseudomonas*. The closest genome is that of *B. thuringiensis* X023 (GenBank GCA_021651035.1) [19,35], with a homology of 99.6% (92.9% alignment coverage). In silico DNA-DNA hybridization (DDH) with *B. thuringiensis* subsp. *galleriae* strain HD-29 showed a 92.13% probability that BTG belongs to the same subspecies [32,36]. The genomes with the highest homology to BTGs are presented in Table 2.

The functional annotation showed that the strain most likely does not contain plasmids, which was confirmed by PlasmidFinder analysis. This suggests that *cry* genes are most likely located in the chromosome, similarly to *B. thuringiensis* subsp. *Berliner* (ATCC 13367) and *B. thuringiensis* HER1410 [37,38]. Considering *cry* genes, *B. thuringiensis* BTG contained seven complete open reading frames (ORFs) encoding the following crystal toxins with insecticidal activity: Cry1Ab35, Cry1Db, Cry1Fb, Cry1Ib, Cry2Ab, Cry8Ea1, and Cry9Ba2, and two partial ORFs, homologous to *cry9Aa* and *cry1Ac* (Table 3 and Table S1).

It should be noted, however, that the sequence annotated as Cry1Ga proved to be identical to that of Cry8Ea1 on protein level (1166 AA). The sequence annotated as Cry9Ea proved to be identical to that of Cry9Ba2 on both DNA (3354 bp) and protein (1117 AA) levels. Cry1Ac was detected as a fragment of only 135 bp, which is not enough to distinguish it from closely related toxins of the same type and first two ranks (Cry1A). The genes encoding pesticidal crystal protein Cry1Fb, cry1Db, cry1Ib, and the partial genes for Cry9Aa and cry1Ca form a cluster in Contig68. Adjacently to *cry* genes one can find genes encoding integrase (possessing 97% similarity with *B. thuringiensis* GR007 plasmid pGR157), and tyrosine recombinase XerS (99% similar to that in the plasmid of *Ps. synxantha* strain 27). However, the genes responsible for plasmid replication were not found in proximity, thus suggesting that the group of *cry* genes in BTG is located in the chromosome (as a possible result of a former recombination event) rather than in autonomous plasmid. In addition, nucleotide sequences with different levels of homology with the unnamed plasmid of *Ps. synxantha* strain 27 were found in nine different BTG contigs (13, 41, 68, 84, 86, 102, 120, 137, and 148).

As far as our current knowledge goes, this toxicological profile appears to be unique to BTG and is not known to exist in any other strain. The BTG genome also bears 107 genes dedicated to defence mechanisms, 116 genes for posttranslational modification, protein turnover, and chaperones, and 66 genes for the biosynthesis of secondary metabolites. Considering the last, in silico analysis by AntiSMASH web service https://antismash.secondarymetabolites.org/ (accessed on 1 May 2023) showed that the BTG genome contains a cluster encoding the synthesis of non-ribosomal antifungal lipopeptide fengycin, metallophore bacillibactin, and siderophore petrobactin, as well as the ribosomally synthesized and post-translationally modified linear azoline-containing peptides (LAPs) with antimicrobial activity [48].

## 3. Discussion

There are numerous patents describing *B. thuringiensis* strains with insecticidal activity and their characteristics. Several US patents protect particular strains (WO2016115476A1, WO1996028031A1), while others protect the discovery of new Cry proteins (US2008020967, US2008040827, US2007245430, US2008016596, US2008020968), or the cloning of the responsible genes in transgenic plants. There are also patents that describe new hybrid insecticidal toxins and their production (US5593881, US5932209, US6780408). Due to the huge interest in the species, 745 genomes of *B. thuringiensis* have been sequenced and assembled to date, as the NCBI database tree contains 603 annotated genomes. However, regardless of the accumulated huge database, the de novo assembled genome of *B. thuringiensis* BTG contains nearly a quarter of genes that are of unknown function. Certain regions of the BTG genome are homologous to the genomes of the strains with widely applied insecticidal activity as *B. thuringiensis* ABTS-1857 (CP083156.1) and *B. thuringiensis* YBT-1520 (CP004858.1) of serovar *kurstaki*. There are also certain differences. For example, ABTS-351 (the active ingredient of DiPel^®^) and YBT-1520 (patent-protected in China) contain genes for Cry1Aa and Cry2Aa toxins, while the BTG strain does not contain such genes. Since *B. thuringiensis* BTG belongs to the subspecies *galleriae* according to ANI estimation, we compared its genome to that of *B. thuringiensis* serovar *galleriae* HD-29 [36]. The similarity between genomes is 90%, with the most significant differences in the encoded crystal toxins. Strain HD-29 contains plasmid-encoded *cry1Aa*, *cry1Ac*, *cry1Ca*, *cry1Ia*, *cry9Ea*, and *vip3Aa*, which are absent in strain BTG. A toxin gene present in the genomes of both strains is *cry2Ab* with 99.6% homology. The nucleotide sequence of *cry1Ab35* of BTG is 100% identical to that of *B. thuringiensis* strain GS36 [35,49]. The toxin is very close (99.97% identity) to a plasmid-encoded toxin of *Pseudomonas synxantha* strain 27 (GenBank CP074079, PRJNA725964). Notably, the same plasmid shows 100% identity with *cry8Ea1* of BTG.

It was recently discovered that a strain of *B. thuringiensis* subsp. *galleriae* (SDS-502), registered in the USA and Canada, produces Cry8Da toxin, which has demonstrated effectiveness against scarab beetles such as *Popillia japonica* [50]. Furthermore, Shrestha et al. [51] reported that strain SDS-502 can be used for biological control of the alfalfa bollworm.

Considering toxins Cry1Ab35, Cry1Db, Cry1Fb, Cry1Ib, Cry2Ab, Cry8Ea1, and Cry9Ba2/Cry9Ea, they have been shown to be effective against at least 10 harmful species from the orders Lepidoptera and Coleoptera. Cry1Ab35 is active against many representatives of Lepidoptera, and one of its most important applications is to control the maize pest *Ostrinia nubilalis* (Hübner), family Pyralidae. Against this insect, only the endophytic fungi of the species *Beauveria bassiana* has been used as a successful biopesticide so far [52]. Cry1Ab35 is a toxin that affects representatives of class *Gastropoda*, which are various types of snails and slugs [53]. Cry1Ab35 and Cry1Fb are effective against *Spodoptera frugiperda* (armyworm), which causes severe damage to crops worldwide [54,55] and very often develops resistance to pesticides [56]. Cry1Fb is toxic to members of the family Noctuidae, which include *Spodoptera ornithogalli* (yellow-striped nightshade) and *Heliothis virescens* (tobacco budworm), which attack tobacco, cotton, alfalfa, tobacco, beans, soybeans, corn, cotton, cabbage, lettuce, and ornamental species such as geranium, hibiscus, pelargonium, and chrysanthemum [57]. Cry1lb toxin is also known to be active against larvae of the cabbage moth *Plutella xylostella* [58], a cruciferous pest that causes USD 2.7 billion worth of damage annually worldwide [59]. Cry2Ab is effective against two cosmopolitan pests, the cotton bollworm *Helicoverpa armigera* [60], and the cabbage bollworm *Trichoplusia ni* [61], affecting over 180 different plant species and capable of developing remarkable resistance to chemical pesticides [57]. Cry8-type toxins are insecticidal to a number of arthropod pests, especially certain scarab beetles (Scarabaeidae) [62]. The Cry8Ea1 protein of Chinese isolate *B. thuringiensis* BT185 is specifically toxic against the larvae of *Holotrichia parallela* (black Asian chafer), a pest that destroys the underground parts of more than 300 species in Europe and Asia, causing massive and irreversible economic losses [63]. Cry9 family toxins have been shown to exhibit toxicity against the large wax moth *Galleria melonella* [64], which is an enemy of honeybees [65]. Additionally, these toxins have also demonstrated efficacy against the beet cutworm, which currently lacks effective biopesticides for control [66]. Cry9Ba2/9Ea (first described in *B. thuringiensis* subsp. *galleriae* HD29) is toxic to the beet armyworm *Spodoptera exigua* and the cabbage bollworm *Trichoplusia ni*, both polyphagous and cosmopolitan pests [67]. There is currently a lack of available data regarding the potency and specificity of Cry9Ba2, making it a very rare toxin with limited information. Cry9Ea is toxic to the larvae of *Cydia pomonella*, the coddling moth, which is a major threat to apples and pears on six continents [68]. So far, in the patent literature, no strains that exhibit combined insecticidal activity against different classes of insects and snails have been described. The specific activities of the known *B. thuringiensis* strains used as biopesticides are compared in Table 4.

The relationship between the production of certain δ-endotoxins and the crystals formed is not fully understood. Some crystal shapes have been related to the synthesis of specific Cry proteins. According to Djenane et al. [74], the expression of *cry4*, *cry10*, or *cry11* genes gives rise to spherical shape crystals, and their respective proteins are known to be active against Diptera [75]. Crystals with a bipyramidal shape result from the accumulation of Cry1 or Cry9 proteins, which are active mainly against Lepidoptera. Cry2 proteins are active against both Lepidoptera and Diptera and form cuboidal crystals [74]. There are also other rare studies on the relationship between crystal morphology and *cry* gene content, for example, Azizoglu et al. [76] cloned the *cry1Ab* gene and showed that it is involved in the formation of bipyramidal crystals. Based on a study of 700 newly isolated strains of *B. thuringiensis* from Qatar, divided into 16 different groups depending on the crystals formed, Nair et al. [22] concluded that the isolates producing bipyramidal and cuboidal crystals carry all the Lepidopteran and Coleopteran specific insecticidal protein-coding genes, and these crystals are formed from the most common Cry1A, Cry1Ia, Cry1B, Cry1D, and Cry2 proteins. This statement partially contradicts the observations of Wanapaisan et al. [77], who demonstrated that Cry1Da forms spherical crystals. Rosas-García linked the presence of proteins of Cry1 and Cry2 classes with bipyramidal and square crystals [78]. In the work of Nair et al. [22], the most common bipyramidal form (characteristic of *Bt* serovar *kurstaki*) was associated with the obligatory presence of a protein of about 130 kDa (corresponding both to Cry1Aa and Cry1Ab). The spherical crystals, on the other hand, with a typical representative *Bt* serovar *israelensis*, also contain a 130 kDa protein core but are formed by a total of 15 proteins of different sizes. The cubic forms of the crystals were composed of a mixture of proteins with molecular weights of 130, 73, 34, 25, and 13 kDa, with the 50−66 kDa proteins being particularly abundantly [79]. Summarizing these data, we can assume that the bipyramidal crystal forms in the newly isolated *B. thuringiensis* PL1, PL3, and PL20, which is due to the presence and expression of the cry1Ab gene encoding a 133.5 kDa protein (Table 3). The cubic crystals of *B. thuringiensis* strain BTG are most likely formed by Cry1Ab35 and Cry2Ab proteins, similar to the quasi-cuboidal shape of Cry1Ab21 crystal protein that was previously found in other *Bt* isolates [80]. The spherical crystals of BTG, however, are not due to the auto-crystallization of proteins of the Cry4 class (since the genome lacks the related genes), but to Cry1Da, as suggested by Wanapaisan et al. [77].

In conclusion, according to the toxin type and specificity reported, *B. thuringiensis* BTG is most likely active mostly against members of orders Lepidoptera and Coleoptera, to a lesser extent orders Diptera and Hemiptera as well as class Gastropoda. The combination of several crystal shapes within an individual *B. thuringiensis* isolate is an indication of the presence of Cry proteins from different families and holds the potential for a spectrum of activity against a broad range of insect pests.

## 4. Materials and Methods

### 4.1. Collection of Soil Samples, Isolation, and Storage of Bacterial Strains

Soil samples were collected from several different geographical regions of Bulgaria (Plovdiv, Sofia, Veliko Tarnovo, Targovishte, Shabla, and the Rhodope Mountains). Notably, the majority of the strains containing *cry* genes (PL1, PL3, PL20, and BTG) were isolated from the Plovdiv region from the *Oryza sativa* rhizosphere (Table 5).

The soil samples were taken with a sterile spatula at a depth of 5 cm from the soil surface. All samples were placed in sterile Falcon tubes and stored at 4 °C until processed.

Soil samples were subjected to treatment in order to isolate strains of microorganisms belonging to the genus *B. thuringiensis*. The thermal shock was used to eliminate all bacteria unable to transform into endospores. One gram of the soil samples was mixed with 10 mL of physiological solution in sterile flasks, followed by incubation on a shaker at 200 rpm for 2 h at 30 °C. Aliquots of 1 mL of the solutions were heat shocked at 80 °C for 10 min and then placed on ice for 5 min. The heat-treated samples were serially diluted in saline and aliquots of each dilution were spread on Petri dishes with NB medium, and incubated at 37 °C for 24–72 h. Colonies that showed typical characteristics of *Bacillus* spp. were sub-cultured and stored at 4 °C for further identification. Long-term storage of the isolates was in liquid media with 20% *v/v* glycerol at −70 °C.

### 4.2. DNA Isolation, 16S rRNA Gene PCR, and Sequencing

For DNA isolation from 24-h cultures of the different strains, GeneMATRIX Bacterial & Yeast Genomic DNA Purification Kit (EURx, Gdansk, Poland) was used. The amplification of the gene encoding the 16S ribosomal RNA was carried out within QB-96 Satellite Gradient Thermal Cycler (LKB Vertriebs GmbH, Vienna, Austria) with universal primers 27F and 1492R, at the following temperature regime: initial denaturation 94 °C for 3.30 min, 35 cycles: 94 °C for 1 min, 58 °C for 45 s, 72 °C for 2 min, and final elongation 72 °C for 7 min. After the visualization of fragments of about 1600 bp in a 1% agarose gel, the PCR products were sent for sequencing. Analysis of the obtained sequences was performed with Chromas LITE version 2.6.6, BLAST, Cap3 (https://doua.prabi.fr/software/cap3, accessed on 25 April 2023), and ClustalW programs.

### 4.3. B. thuringiensis BTG Genome: Sequencing, Assembly, and Bioinformatics Analysis

De novo sequenced genome was registered in NCBI GenBank under the following WGS accession: BioProject PRJNA977062, BioSample SAMN35448569, acc. number JASNQP000000000.

All procedures on genome library preparation, WGS, and genome annotation were accomplished by the company Macrogen Inc. (Seoul, Republic of Korea). The genome library was constructed using TruSeq DNA PCR-Free Kit; the sequencing was done by the Illumina HiSeq 2000 Sequencing System, read length 151. Total read bases were 2,251,811,962. The genome coverage was 91.85x.

Platanus-allee v2.2.2, de novo haplotype assembly method using De Bruijn Graph (DBG) algorithm [80] generated 153 contigs with N50 = 58,552 (estimated genome size 6,275,416), with a sequencing depth of 163. The assembled genome was validated using Benchmarking Universal Single-Copy Orthologs (BUSCO) analysis [81] based on evolutionarily informed expectations of gene content from near-universal single-copy orthologs, as the used lineage was “bacteria_odb10” (number of genomes: 4085, number of BUSCOs: 124). Complete and single-copy BUSCOs were 119 (95.97%), while complete and duplicated BUSCOs were 5 (4.03%). Fragmented and missing BUSCOs were 0.0%.

The functional annotation was conducted by InterProScan and Eggnog DB, and the Type (Strain) Genome Server (TYGS) https://tygs.dsmz.de/ (accessed on 13 January 2023) was used for *B. thuringiensis* serovar elucidation [82]. The possible presence of plasmids was searched with the program PlasmidFinder set for Gram-positive bacteria [83] and did not detect plasmids. AntiSMASH 7.0 platform predicted secondary metabolites synthesis [84].

### 4.4. PCR Screening for Cry Toxins Encoding Genes

The genes encoding 12 different Cry toxins were selected for potential detection. These toxins are potential pests against six orders (Lepidoptera, Coleoptera, Diptera, Hemiptera, and Hymenoptera) and one class (Gastropoda) from three different phyla (Arthropoda, Nematoda, and Mollusca) according to the literature [8,85,86] and the NCBI GenBank database. Primers were designed to amplify fragments from 559 to 2181 bp (Table 6). Sequencing of the PCR fragments was performed by Macrogen Inc. (Amsterdam, The Netherlands).

PCR reactions consisted of 50 ng DNA template, 0.4 μM primers, Premix Ex Taq HotStart Version (Clontech Laboratories, Inc., A Takara Bio Company, Mountain View, CA, USA), and sterile water with a final volume of 25 μL. Between initial denaturation for 3 min and 30 s at 98 °C and final elongation for 5 min at 72 °C, the following temperature profile was used for 35 cycles: 10 s denaturation at 98 °C, 30 s annealing at the most appropriate temperature in a range from 43.3 to 55.9 °C (as determined by gradient PCR), and 2 min elongation at 72 °C.

The PCR fragments were visualized on 1% agarose gel and used for sequencing.

### 4.5. Scanning Electron Microscopy (SEM)

Spores and crystals of *B. thuringiensis* PL1, PL3, PL20, and BTG were observed by scanning electron microscopy after 72 h of incubation in a spore-forming medium containing (NH_4_)_2_SO_4_, 1 g/L; K_2_HPO_4_, 4.15 g/L; KH_2_PO_4_, 3.4 g/L; Salt solution, 3 ml/L with content as previously described [87]; sucrose, 10 g/L, and soybean meal, 60 g/L. Bacterial biomass was harvested by centrifugation and washed with cacodylate buffer pH (7.2). Pelleted cells were fixed with 4% glutaraldehyde for 2 h, followed by post-fixation with 1% OsO_4_ for 1 h at 4 °C. After washing three times, dehydration follows in graded ethanol series through 15 min time intervals. The final stage was mounting on scanning electron microscopy holders and sputtering with gold in a vacuum evaporator (Edwards, CA, USA). The observations were made on Lyra/Tescan scanning electron microscope (Riga, Latvia).

## 5. Conclusions

The isolation and genetic characterization of new *B. thuringiensis* strains containing *cry* toxin genes were reported. Seven strains contained various genes encoding δ-endotoxins. Four strains had the genetic ability to produce more than three different Cry toxins, as demonstrated by PCR analysis and whole genome sequencing. SEM observation of the formed crystalline structures showed their correlation with the detected genes. *B. thuringiensis* BTG genome mining showed a particularly rich and unique combination of *cry* genes, allowing for the future development of industrial preparations against multiple pests.

## Figures and Tables

**Table 1:** PCR screening for *cry* genes in *B. thuringiensis* strains.

Strain	*cry* Genes		
	Sequenced	PCR Product	Not Presented
BTG	*cry1Ab35*, *cry2Ab*, *cry9Ba2*	*cry* *7Aa*	*cry1Aa*, *cry2Aa*, *cry3Aa*, *cry5A*, *cry8Ba2*, *cry9Ca*, *cry9Da*, *cry11A*, *cry15A*, *cry22A*
PL1	*cry* *1Ab*	*cry7Aa*, *cry8Ba2*	*cry1Aa*, *cry2Aa*, *cry3Aa*, *cry5A*, *cry9Ca*, *cry9Da*, *cry11A*, *cry15A*, *cry22A*
PL3	*cry* *1Ab*	*cry7Aa*, *cry8Ba2*	
PL20	*cry* *1Ab*	*cry7Aa*, *cry8Ba2*	
30	-	*cry* *1Ab*	*cry1Aa*, *cry2Aa*, *cry3Aa*, *cry5A*, *cry7Aa*, *cry8Ba2*, *cry9Ca*, *cry9Da*, *cry11A*, *cry22A*
40	-	*cry* *1Ab*	
38	-	*cry* *11A*	

**Table 2:** Summarized BLAST comparison of de novo sequenced genome of *B. thuringiensis* strain *BTG* with *B. thuringiensis* genomes (NCBI GenBank database).

Sequence (Accession Number)	Species/Strain	BTG Genome Contigs (Number)	Total Length with >99% Homology (bp)
CP045585.1	*B. thuringiensis* strain X023	66	1,958,310
CP010089.1	*B. thuringiensis* serovar *galleriae* HD-29	22	750,128
CP004858.1	*B. thuringiensis* serovar *kurstaki* YBT-1520	5	442,012
CP083156.1	*B. thuringiensis* ABTS-1857	2	391,953
CP004069.1	*B. thuringiensis* serovar *kurstaki* HD73	4	328,551
CP013055.1	*B. thuringiensis* YWC2-8	6	289,587

**Table 3:** *Cry* genes encoding crystal δ-toxins in *B. thuringiensis* BTG.

Name	Gene (bp)	Cry Protein (Amino Acids)	Cry Protein (kDa) ^†^	Gene Homology (GenBank Accession No.)	Reference
*cry1* *Ab* *35*	3546	1181	133.5	KT692985 (100%)	[39]
*cry* *1Db*	3483	1160	131.0	AF358862 (99%)	[40]
*cry* *1Fb*	3525	1174	133.3	AF336114 (100%)	[41]
*cry* *8Ea1*	3501	1166	132.1	WP_033698569 (100%)	[42]
*cry* *1Ib*	2160	719	81.5	BTU07642, EU677422 (99.9%)	[43]
*cry* *2Ab*	1902	633	70.7	ON508057 (100%)	[44]
*cry* *9Ba2*	3354	1117	126.4	GU299522 (100%)	[45]
*cry9Aa* *	276	91	-	JX174110 (100%)	[46]
*cry1Ac* *	135	44	-	CP076540 (100%)	[47]

* Partial sequence; ^†^ computed molecular weight.

**Table 4:** Predicted insecticidal activity of the Cry-proteins of *B. thuringiensis* isolated in Bulgaria in comparison with literature data.

Cry Toxins	*B. thuringiensis* Serovar	Strain	Target	Reference
1Aa, 1Ab, 1Ac, 2Aa, 2Ab	*kurstaki*	ABTS-351	Lepidoptera	[69]
1Aa, 1Ab, 1Ac, 2Aa	*kurstaki*	HD1	Lepidoptera	[70]
1Ac	*kurstaki*	HD73	Lepidoptera	[70]
1Aa, 1Ba, 1Ca, 1Da	*aizawai*	HD137	Lepidoptera	[71]
1Aa, 2Ba, 4Aa, 4Ba, 10Aa	*israelensis*	-	Diptera	[72]
3Aa	*tenebrionis*	-	Coleoptera	[73]
1Aa, 1Ac, 1Ca, 1Da, 1Ia, 2Ab, 9Ea	*galleriae*	HD-29	Lepidoptera	[36]
8Da	*galleriae*	SDS-502	Coleoptera	[51]
1Ab, 1Db, 1Fb, 1Ib, 2Ab, 8Ea1, 9Ba2	*galleriae*	BTG	Lepidoptera Coleoptera	This study
1Ab, 7Aa, 8Ba2	*-*	PL1, PL3, PL20	Lepidoptera Coleoptera	This study
1Ab	*-*	30, 40	Lepidoptera	This study
11A	*-*	38	Lepidoptera	This study

**Table 5:** Isolation of *B. thuringiensis* strains: geographical location of sampling sites in Bulgaria.

Species/Strain	Source	Region/Town	Coordinates
*Bt* serovar *galleriae* BTG	Loam soil, rice rhizosphere	Plovdiv, Trud	N 42°14′, E 24°44′
*B. thuringiensis* PL1	Loam soil, rice rhizosphere	Tsaratsovo	N 42°12′, E 24°41′
*B. thuringiensis* PL3	Waterlogged clay soil	Gelemenovo	N 42°16′, E 24°19′
*B. thuringiensis* PL20	Loam soil, rice rhizosphere	Kurtovo Konare	N 42°06′, E 24°30′
*B. thuringiensis* 30	Loam soil, maize field	Targovishte	N 43°16′, E 26°23′
*B. thuringiensis* 38	Loam soil	Veliko Tarnovo	N 43°04′, E 25°37′
*B. thuringiensis* 40	Silt/Sandy soil	Shabla	N 43°37′, E 28°31′

**Table 6:** *Cry* genes selected for PCR screening in *B. thuringiensis* strains.

Endotoxin	Target	Gene (bp)	PCR Fragment (bp)	Primers (5′-3′)	Accession Number (NCBI)
1Aa	Lepidoptera Diptera	3543	704	F: TCAAATTATGATAGTCGAAG R: CCAAGATTAGTAGATTTTGTTA	KM924540
1Aa ^1^	Lepidoptera Diptera	4222	951	F: TGGCTCTGGAACTTCTGTCG R: TTCGGCTCTCCACACTTTCC	M11250
1Ab	Lepidoptera Gastropoda	5139	1132	F: CTCCTGTAGGGTTTTCGGGG R: CTCCAGCCACGGTCTAGTTG	AF358861
2Aa/2Ab	Lepidoptera Diptera Hemiptera	1902	559	F: TAGTGGACCACAGCAGACAC R: TACCAAATAGGCCCGTGCTC	MK184464 JN226103
3Aa	Coleoptera Hemiptera Hymenoptera	2983	1265	F: ATAGCCAGGGGCGGATAAGA R: TGCCCCGTCTAAACTGAGTG	AJ237900
5A	Hymenoptera Rhabditida	4377	743	F: CACCACCAGGATTGTCTCCAT R: CGTCCAATTGGCTGCATCTT	EF219060
7Aa ^2^	Coleoptera	3417	1764	F: CCCGATCTTTTCTTGGACGC R: ATTCCTATCTTGCGCGCTGT	MK840959
8Ba2 ^3^	Coleoptera	3510	1093	F: TTTAACGGACCGCATCGGAA R: TGCGTCTCCTGACAAAGGTC	MZ355710
9Ca ^4^	Lepidoptera	5772	1954	F: GGATGGGGATTCACACAGGG R: GTGTTTGAGCCGCTTCACAG	Z37527
9Da/9Ba2	Coleoptera Lepidoptera	3456 3495	2181	F: TGCACAAGCAGCAAACCTTC R: TCACCCGATAATGGCCCAAC	GQ249295 GU299522
11Aa	Diptera Hemiptera	1941	1476	F: AACCTACTATTGCGCCAGCA R: TACTGCCGTCTGTTGCTTGA	MK184471
15A ^5^	Lepidoptera	2259	1628	F: GGGGGTGGTAAGCCTGAAAT R: CTCTCGTCGTTGCTGTTCCT	M76442
22A	Lepidoptera Coleoptera Hymenoptera	2169	1631	F: TAGGCGATTCGTTTGGGCAT R: GAAAACTGGCGGCTCTTCTG	EU715020

^1^ High level of homology between cry1Aa/1Ab > 93%; ^2^ Toxin active against Colorado beetle larvae [86]; ^3^ Toxin active against Colorado beetle imago; ^4^ Broad-spectrum toxins against several Lepidopteran families such as Pyralidae, Plutellidae, Sphingidae, and Noctuidae. Toxic against the larvae of moths of the Noctuidae family [86]; ^5^ An unidentified novel toxin identical in sequence to *cry15* (MN557412).

## Data Availability

The *B. thuringiensis* serovar *galleriae* whole genome shotgun (WGS) project has the project accession JASNQP000000000. This version of the project (01) has the accession number JASNQP010000000 and consists of sequences JASNQP010000001-JASNQP010000153. All genome data are available in the NCBI GenBank following a patent deposit of *B. thuringiensis* strain BTG (NBIMCC №9095).

## Supplementary Materials

The following supporting information can be downloaded at: https://www.mdpi.com/article/10.3390/ijms241311137/s1**.**
