# Supplementary material for: Multiple cry Genes in Bacillus thuringiensis Strain BTG Suggest a Broad-Spectrum Insecticidal Activity
Source: Int J Mol Sci. 2023 Jul 6;24(13):11137. doi: 10.3390/ijms241311137 (PMC10342485; doi:10.3390/ijms241311137)
Supplement: Supplementary file 1 [file ijms-24-11137-s001.zip › ijms-2462798-supplementary.pdf]

Table S1. Nucleotide sequences of *cry* genes found in the genome of *Bacillus thuringiensis* subsp. *galleriae* strain BTG and the deduced amino acid sequences of Cry δ-endotoxins.

Cry gene	**Length (bp)**	**Sequence (5′-3′)**	**Amino acids**	**Sequence**
cry1Ab35	**3546**	atggataacaatccgaacatcaatgaatgcattcctt<br>ataattgtttaagtaaccctgaagtagaagtattagg<br>tggagaaagaatagaaactggttacaccccaatcg<br>atatttccttgtcgctaacgcaatttcttttgagtgaatt<br>tgttcccggtgctggatttgtgttaggactagttgat<br>ataatatggggaattttggtccctctcaatgggacg<br>catttcttgtacaaattgaacagttaattaaccaaag<br>aatagaagaattcgctaggaaccaagccatttctag<br>attagaaggactaagcaatctttatcaaatttacgca<br>gaatcttttagagagtgggaagcagatcctactaat<br>ccagcattaagagaagagatgcgtattcaattcaat<br>gacatgaacagtgcccttacaaccgctattcctcttt<br>tgcagttcaaaattatcaagttcctcttttatcagtata<br>tgttcaagctgcaaatttacatttatcagttttgagag<br>atgtttcagtgtttggacaaaggtggggatttgatg<br>ccgcgactatcaatagtcgttataatgatttaactag<br>gcttattggcaactatacagatcatgctgtacgctgg<br>tacaatacgggattagagcgtgtatggggaccgg<br>attctagagattggataagatataatcaatttagaag<br>agaattaacactaactgtattagatatcgtttctctatt<br>tccgaactatgatagtagaacgtatccaattcgaac<br>agtttcccaattaacaagagaaatttatacaaaccca<br>gtattagaaaattttgatggtagttttcgaggctcgg<br>ctcagggcatagaaggaagtattaggagtccacat<br>ttgatggatatacttaacagtataaccatctatacgg<br>atgctcatagaggagaatattattggtcagggcatc<br>aaataatggcttctcctgtagggttttcggggccag<br>aattcacttttccgctatatggaactatgggaaatgc<br>agctccacaacaacgtattgttgctcaactaggtca<br>gggcgtgtatagaacattatcgtccactttatataga<br>agaccttttaatatagggataaataatcaacaactat<br>ctgttcttgacgggacagaatttgcttatggaacctc<br>ctcaaatttgccatccgctgtatacagaaaaagcgg<br>aacggtagattcgctggatgaaataccgccacaga<br>ataacaacgtgccacctaggcaaggatttagtcatc<br>gattaagccatgtttcaatgtttcgttcaggctttagt<br>aatagtagtgtaagtataataagagctcctatgttct<br>cttggatacatcgtagtgctgaatttaataatataatt<br>ccttcatcacaaattacacaaatacctttaacaaaatc<br>tactaatcttggctctggaacttctgtcgttaaagga<br>ccaggatttacaggaggagatattcttcgaagaact<br>tcacctggccagatttcaaccttaagagtaaatatta<br>ctgcaccattatcacaaagatatcgggtaagaattc<br>gctacgcttctaccacaaatttacaattccatacatca<br>attgacggaagacctattaatcaggggaattttca<br>gcaactatgagtagtgggagtaatttacagtccgg<br>aagctttaggactgtaggttttactactccgtttaactt	**1181**	MDNNPNINECIPYNCLSNPEV<br>EVLGGERIETGYTPIDISLSLTQF<br>LLSEFVPGAGFVLGLVDIIWGIF<br>GPSQWDAFLVQIEQLINQRIEE<br>FARNQAISRLEGLSNLYQIYAES<br>FREWEADPTNPALREEMRIQF<br>NDMNSALTTAIPLFAVQNYQV<br>PLLSVYVQAANLHLSVLRDVS<br>VFGQRWGFDAATINSRYNDLT<br>RLIGNYTDHAVRWYNTGLERV<br>WGPDSRDWIRYNQFRRELTLT<br>VLDIVSLFPNYDSRTYPIRTVSQ<br>LTREIYTNPVLENFDGSFRGSA<br>QGIEGSIRSPHLMDILNSITIYT<br>DAHRGEYYWSGHQIMASPVG<br>FSGPEFTFPLYGTMGNAAPQQ<br>RIVAQLGQGVYRTLSSTLYRRP<br>FNIGINNQQLSVLDGTEFAYGT<br>SSNLPSAVYRKSGTVDSLDEIPP<br>QNNNVPPRQGFSHRLSHVSM<br>FRSGFSNSSVSIIRAPMFSWIHR<br>SAEFNNIIPSSQITQIPLTKSTNL<br>GSGTSVVKGPGFTGGDILRRTS<br>PGQISTLRVNITAPLSQRYRVRI<br>RYASTTNLQFHTSIDGRPINQG<br>NFSATMSSGSNLQSGSFRTVGF<br>TTPFNFSNGSSVFTLSAHVFNS<br>GNEVYIDRIEFVPAEVTFEAEY<br>DLERAQKAVNELFTSSNQIGL<br>KTDVTDYHIDQVSNLVECLSD<br>EFCLDEKKELSEKVKHAKRLS<br>DERNLLQDPNFRGINRQLDRG<br>WRGSTDITIQGGDDVFKENYV<br>TLLGTFDECYPTYLYQKIDESK<br>LKAYTRYQLRGYIEDSQDLEIY<br>LIRYNAKHETVNVPGTGSLWP<br>LSAPSPIGKCGEPNRCAPQLE<br>WNPDLDCSCRDGEKCAHHSH<br>HFSLDIDVGCTDLNEDLGVW<br>VIFKIKTQDGHARLGNLEFLEE<br>KPLVGEALARVKRAEKKWRD<br>KREKLEWETNIVYKEAKESVD<br>ALFVNSQYDRLQADTNIAMIH<br>AADKRVHSIREAYLPELSVIPG<br>VNAAIFEELEGRIFTAFSLYDAR<br>NVIKNGDFNNGLSCWNVKG<br>HVDVEEQNNHRSVLVVPEWE<br>AEVSQEVRVCPGRGYILRVTAY<br>KEGYGEGCVTIHEIENNTDEL<br>KFSNCVEEEVYPNNTVTCNDY<br>TATQEEYEGTYTSRNRGYDGA<br>YESNSSVPADYASAYEEKAYTD<br>GRRDNPCESNRGYGDYTPLPA<br>GYVTKELEYFPETDKVWIEIGE<br>TEGTFIVDSVELLLMEE

ttcaaatggatcaagtgtatttacgttaagtgctcat
gtcttcaattcaggcaatgaagtttatatagatcgaa
ttgaatttgttccggcagaagtaacctttgaggcag
aatatgatttagaaagagcacaaaaggcggtgaat
gagctgtttacttcttccaatcaaatcgggttaaaaa
cagatgtgacggattatcatattgatcaagtatccaa
tttagttgagtgtttatctgatgaattttgtctggatga
aaaaaaagaattgtccgagaaagtcaaacatgcg
aagcgacttagtgatgagcggaatttacttcaagat
ccaaactttagagggatcaatagacaactagaccg
tggctggagaggaagtacggatattaccatccaag
gaggcgatgacgtattcaaagagaattacgttacg
ctattgggtacctttgatgagtgctatccaacgtattt
atatcaaaaaatagatgagtcgaaattaaaagccta
tacccgttaccaattaagagggtatatcgaagatag
tcaagacttagaaatctatttaattcgctacaatgcca
aacacgaaacagtaaatgtgccaggtacgggttcc
ttatggccgctttcagccccaagtccaatcggaaaa
tgtggagaaccgaatcgatgcgcaccacaacttga
atggaatccagatctagattgttcctgcagagacgg
agaaaaatgtgcccatcattcccatcatttctccttgg
acattgatgttggatgtacagacttaaatgaggactt
aggtgtatgggtgatattcaagattaagacgcaag
atggccatgcaagactaggaaatctagaatttctcg
aagagaaaccattagtaggagaagcactagctcgt
gtgaaaagagcggagaaaaaatggagagacaa
acgtgaaaaattggaatgggaaacaaatattgttta
taaagaggcaaaagaatctgtagatgctttatttgta
aactctcaatatgatagattacaagcggataccaac
atcgcgatgattcatgcggcagataaacgcgttcat
agcattcgagaagcttatctgcctgagctgtctgtga
ttccgggtgtcaatgcggctatttttgaagaattaga
agggcgtattttcactgcattctccctatatgatgcga
gaaatgtcattaaaaatggtgattttaataatggctt
atcctgctggaacgtgaaagggcatgtagatgtag
aagaacaaaacaaccaccgttcggtccttgttgttcc
ggaatgggaagcagaagtgtcacaagaagttcgt
gtctgtccgggtcgtggctatatccttcgtgtcacag
cgtacaaggagggatatggagaaggttgcgtaac
cattcatgagatcgagaacaatacagacgaactga
agtttagcaactgtgtagaagaggaagtatatcca
aacaacacggtaacgtgtaatgattatactgcgact
caagaagaatatgagggtacgtacacttctcgtaat
cgaggatatgacggagcctatgaaagcaattcttct
gtaccagctgattatgcatcagcctatgaagaaaaa
gcatatacagatggacgaagagacaatccttgtga
atctaacagaggatatggggattacacaccactac
cagctggctatgtgacaaaagaattagagtacttcc
cagaaaccgataaggtatggattgagatcggaga
aacggaaggaacattcattgtggatagcgtggaat
tactccttatggaggaatag

cry1Db	**3483**	atggatataaatcatcaaaatcaatgtataccctaca<br>attgtttaagtaatcctgatgcgatattactagatgc<br>ggaaaggctagaaacaggaaacaccgtagcaga<br>catttcattagggcttattaactttctatattctaatttg<br>taccaggcggagggtttatagtaggtttactagaat<br>taatatgggggtttgtggggccctcgcaatgggag<br>atttttttagctcaaatagagcaattgattagtcaaag<br>aatagaagaatttgctaggaaccaagcaatttctag<br>attagaaggattaagcaataattatgagatttataca<br>gaaacgtttcgggcgtgggaaaaggatccgtctaa<br>tcctgcattgagagaagagatgcgtacccaatttaa<br>tgtcatgaatagtgctctcatagcagctattcctctttt<br>aagagttcgtaattatgaagttgctcttttatccgttta<br>tgttcaggcagcaaatttacacctatctgtgttaaga<br>gatgtttcagtttacggtcagagatggggctttgac<br>ccagcgactgtcaatagtcgttatagcgatttaacta<br>ggcttattcatgtttatactgatcattgtgtggatacg<br>tataatgatgggttaaaaaatttagagggatctcga<br>ttgtcagattgggttgtatataatcgctttcgaagac<br>gattaacaatttctgtattagatattatagctttttccc<br>aaattatgatatagaagcgtatccaatacaaacagc<br>tagtcaattgacacgagaagtctatctggatttacct<br>tttgttaatgaaactctatcccctccagcaagctatcc<br>aactttctcagctgctgaaagtgctataattagaagt<br>cctcatttagtagacttttaaatagctttaccatttata<br>cagatagtctggcaagttatgcatattggggaggg<br>cacttggtaaattctttccgcacaggaactactacta<br>atttgataagatcccctctatatggaagggaaggaa<br>atacagagcgtcccgtaactatttccgcatcacctag<br>cgtaccaatatttagaacactttcatatttactggtct<br>taacaataataatcctgtagctggaatcgagggag<br>tggaattccaaaatactataagtagaagtatctatcg<br>taaaagcggtccaatagattcttttagtgaattacca<br>cctcaagatgtcagtgtatctcctgcaattgggtata<br>gtcatcgtttatgccatgcaacattttagaacggatt<br>agtggaccaagaatagcaggcaccgtatttcttgg<br>acacaccgtagtgctagtcctattaatgaagtaagt<br>ccatctagaattacacaaattccatgggtaaaggcg<br>catactcttgcgtctggtgcctctgtcattaaaggtcc<br>tggatttacaggcggagatattctgactaggaatag<br>tatgggcgacctgggggccttacgagtaacctttac<br>aggaagattaccacaaagttattatatacgtttccgtt<br>atgcttcggtagcaaataggagtggtacatttagat<br>attcacagccaccttcgtatggaatttcatttccaaaa<br>actatggacgcaggtgaagcactaacatctcgttcg<br>ttcgctcatacaacactcttcactccaataacctttca<br>cgagctcaagaagaatttgatctatacatccaatcg<br>ggtgtttatatagatcgaattgaatttataccggtag<br>atgcaacttttgaatcagaaattaatttagaaagagc<br>gcaaaaggcggtgaatgccctgtttacttctacaaa<br>ccaactagggcttaaaacagatgtgacggattatca	**1160**	MDINHQNQCIPYNCLSNPDAI<br>LLDAERLETGNTVADISLGLIN<br>FLYSNFVPGGGFIVGLLELIWG<br>FVGPSQWEIFLAQIEQLISQRIE<br>EFARNQAISRLEGLSNNYEIYT<br>ETFRAWEKDPSNPALREEMRT<br>QFNVMNSALIAAIPLLRVRNY<br>EVALLSVYVQAANLHLSVLRD<br>VSVYGQRWGFDPATVNSRYSD<br>LTRLIHVYTDHCVDTYNDGLK<br>NLEGSRLSDWVVYNRFRRRLTI<br>SVLDIIAFFPNYDIEAYPIQTAS<br>QLTREVYLDLPFVNETLSPPAS<br>YPTFSAAESAIIRSPHLVDFLNS<br>FTIYTDSLASYAYWGGHLVNSF<br>RTGTTTNLIRSPLYGREGNTER<br>PVTISASPSVPIFRTLSYFTGLN<br>NNNPVAGIEGVEFQNTISRSIY<br>RKSGPIDSFSELPPQDVSVSPAI<br>GYSHRLCHATFLERISGPRIAG<br>TVFSWTHRSASPINEVSPSRITQ<br>IPWVKAHTLASGASVIKGPGF<br>TGGDILTRNSMGDLGALRVTF<br>TGRLPQSYYIRFRYASVANRSG<br>TFRYSQPPSYGISFPKTMDAGE<br>ALTSRSFAHTTLFTPITFSRAQE<br>EFDLYIQSGVYIDRIEFIPVDATF<br>ESEINLERAQKAVNALFTSTN<br>QLGLKTDVTDYHIDQVSNLVE<br>CLSDEFCLDEKRELSEKVKHA<br>KRLSDERNLLQDPNFRGINRQ<br>PDRGWRGSTDITIQGGDDVFK<br>ENYVTLTGTFDECYPTYLYQKI<br>DESKLKAYTRYQLRGYIEDSQD<br>LEIYLIRYNAKHEIVNVPGTGS<br>LWPLSVQSPIGKCGEPNRCAP<br>HLEWNPDLDCSCRDEEKCAH<br>HSHHFSLDIDVGCTDLNEDLG<br>VWVIFKIKTQDGHARLGNLEF<br>LEEKPLVGEALARVKRAEKKW<br>RDKREKLELETNIVYKEAKESV<br>DALFVNSQYDQLQADTNIAMI<br>HAADKRVHSIREAYLPELSVIP<br>GVNAGIFEELEGRIFTAYSLYD<br>ARNVIKNGDFNNGLSCWNVK<br>GHVDVEEQNNHRSVLVVPEW<br>EAEVSQEVRVCPGRGYILRVTA<br>YKEGYGEGCVTIHEVDNNTDE<br>LKFSNCEKEQVYPGNTVACND<br>YNKNHGANACSSRNRGYDES<br>YESNSSIPADYAPVYEEEAYTD<br>GQRGNPCEFNRGHTPLPAGY<br>VTAELEYFPETDTVWVEIGETE<br>GTFIVDSVELLLMEE

		tattgatcaggtatccaatctagttgaatgtttatcgg<br>atgaattttgtctggatgaaaagagagaattgtccg<br>agaaagttaaacatgcgaagcgactcagtgatga<br>gcggaatttacttcaagatccaaacttcagagggat<br>caatagacaaccagaccgtggctggagaggaagt<br>acggatattaccatccaaggaggagatgacgtatt<br>caaagagaattacgtcacactaacaggtacctttga<br>tgagtgttatccaacgtatttatatcaaaaaatagat<br>gagtcaaaattaaaagcctacactcgttatcaattaa<br>gagggtacatcgaagatagtcaagacttagaaatc<br>tatttgatccgttacaatgcaaaacacgaaatagta<br>aatgtgccaggcacgggttccttatggccgctttca<br>gtccaaagtccaatcggaaagtgtggagaaccga<br>atcgctgcgcgccacaccttgaatggaatcctgatc<br>tagattgttcctgcagagacgaggaaaaatgtgcc<br>catcattcgcatcatttctccttggacattgatgttgga<br>tgtacagacttaaatgaggacttaggtgtatgggtg<br>atattcaagattaagacgcaagatgggcacgcaag<br>actagggaatctagagtttctcgaagagaaaccatt<br>agtaggagaagcactagctcgtgtgaaaagagcg<br>gagaaaaaatggagagacaaacgtgaaaaattg<br>gaattggaaacaaatatcgtttataaagaggcaaa<br>agaatctgtagatgctttatttgtaaactctcaatatg<br>atcaattacaagcggatacgaatattgccatgattca<br>tgcggcagataaacgtgttcatagcattcgagaag<br>cgtatcttccagagttatctgtaattccgggtgtaaat<br>gcgggcattttgaagaattagagggacgtatttc<br>acagcctactctctatatgatgcgagaaatgtcatta<br>aaaatggtgatttcaataatggcttatcatgctggaa<br>cgtgaaagggcatgtagatgtagaagaacaaaac<br>aaccaccgttcggtccttgttgttccggaatgggaa<br>gcagaagtgtcacaagaagttcgtgtctgtccggg<br>tcgtggctatatccttcgtgtcacagcgtacaaaga<br>gggatatggagaaggctgcgtaactattcatgaag<br>tcgataataatacagacgaattgaagtttagcaact<br>gtgagaaagaacaagtatatccaggtaatacggta<br>gcatgtaatgattataataagaatcacggtgcgaat<br>gcatgtagttctcgtaatcgtggatatgacgaatctt<br>atgaaagtaattcttccataccagctgattatgcacc<br>ggtttatgaagaagaagcgtatacagatggacaa<br>agagggaatccttgtgaatttaacagagggcatac<br>accattaccagctggttatgtgacagcagagttaga<br>gtacttcccagaaacggatacagtatgggttgagat<br>tggagaaacggaaggaacatttatcgtggacagt<br>gtggaattactccttatggaggaatag		
cry1Fb	**3525**	atgaagaataacattcaaaatcaatgcgtaccttac<br>aattgtttaagtaatcctgaagtagaaatattaagtg<br>aagaaagaagtactggcagattaccgttagatatat<br>ccttgtcgcttacacgtttccttttgagtgaatttgttcc<br>aggtgtgggagttgcgtttggattatttgatttaatat<br>ggggttttataactccttctgaatggagtttatttctttt	**1174**	MKNNIQNQCVPYNCLSNPEV<br>EILSEERSTGRLPLDISLSLTRFL<br>LSEFVPGVGVAFGLFDLIWGFI<br>TPSEWSLFLLQIEQLIEQRIETLE<br>RNRAITTLRGLADSYEVYLEAL<br>REWEENPNNAQLREDVRIRFA<br>NTDDALITAINNFTLTSFEIPLL<br>SVYVQAANLHLSLLRDAVSFG

	acagattgaacaactgattgaacaaagaattgaaa<br>cattggaaaggaaccgggcaattactacattacga<br>gggttagcggatagctatgaagtttaccttgaggca<br>ctaagagagtgggaagaaaatcctaataatgcaca<br>attaagggaagatgtgcgtattcgatttgctaataca<br>gacgacgctttaataacagcaataaataattttacac<br>ttacaagttttgaaatccctcttttatcggtctatgttca<br>agcggcgaatctacatttatcactattaagagatgct<br>gtatcgtttgggcagggttgggggctggatatagc<br>tactgttaataatcattataatagattaataaatcttat<br>tcatagatatacggaacattgtttggacacatacaat<br>caaggattagaaaacttaagaggtactaatactcg<br>acaatggtcaagattcaatcagtttaggagagagtt<br>aacattgactgtattagatatcgttgctcttttccgaa<br>ctacgatgctagagcatatccaattcaaacgtcatcc<br>caattaacaagggaaatttatacaagttcagtaattg<br>aagattctccagtttctgctaatatacctaatggtttta<br>atagagcggaatttggagttagaccgccccatctta<br>tggactttatgaattctttgtttgtaactgcagagact<br>gttagaagtcaaactgtgtggggaggacacttagtt<br>agttcacgaaatacggctggtaaccctataaatttcc<br>ctatttatgggatcttcaatcctggtggcgccatttgg<br>attgcagatgaggatccacgtccttttatcggacatt<br>atcagatcctgtttttgtccgaggaggatttgggaat<br>cctcattatgtacttgggcttaggggagtagcatttc<br>aacaaactggtacgaaccacacccgaacatttaga<br>aatagtgggaccatagattctctagatgaaatccca<br>cctcaggataatagtggggcaccttggaatgattat<br>agtcatgtattaaatcatgttacatttgtaaggtggc<br>ctggtgagattgcaggaagtgattcatggagagcg<br>ccaatgttttcttggacacaccgtagtgcagatcgta<br>caaatatcattaatccaaatataattacacaaatacct<br>gctgtaaaagcacacaatcttcattcgggttctacgg<br>ttgttagaggacccgggtttacaggtggtgatctctt<br>acgaagaacgaatactggtacatttgcagatataa<br>gagtaaatattactgggccattatctcaaagatatcg<br>tgtaagaattcgctatgcttctacgacagatttacaat<br>ttttcacgagaatcaatggaacttctgtaaatcaagg<br>taatttccaaagaactatgaatagagggggtaattt<br>agaatctggaaactttaggactgcaggatttagtac<br>gccttttagtttttcaaatgcgcaaagtacattcacatt<br>gggtactcaggctttttcaaatcaggaagtttatata<br>gatcgaattgaatttgtcccggcagaagtaacattc<br>gaggcagaatctgatttagaaagagcgcaaaagg<br>cggtgaatgccctgtttacttctacaagccaactagg<br>gctaaaaacaaatgtaacgggttaccatattgatca<br>agtgtccaatttagttgcgtgtttatcggatgaatttt<br>gtctggatgaaaagagagaattgtccgagaaagtt<br>aaacatgcgaagcgactcagtgataagcggaattt<br>acttcaagatccaaacttcagagggatcaataggc<br>aaccagaccatggctggagaggaagtacggatat	QGWGLDIATVNNHYNRLINLI<br>HRYTEHCLDTYNQGLENLRG<br>TNTRQWSRFNQFRRELTLTVL<br>DIVALFPNYDARAYPIQTSSQL<br>TREIYTSSVIEDSPVSANIPNGF<br>NRAEFGVRPPHLMDFMNSLF<br>VTAETVRSQTVWGGHLVSSRN<br>TAGNPINFPIYGIFNPGGAIWI<br>ADEDPRPFYRTLSDPVFVRGGF<br>GNPHYVLGLRGVAFQQTGTN<br>HTRTFRNSGTIDSLDEIPPQDN<br>SGAPWNDYSHVLNHVTFVRW<br>PGEIAGSDSWRAPMFSWTHRS<br>ADRTNIINPNIITQIPAVKAHN<br>LHSGSTVVRGPGFTGGDLLRR<br>TNTGTFADIRVNITGPLSQRYR<br>VRIRYASTTDLQFFTRINGTSV<br>NQGNFQRTMNRGGNLESGNF<br>RTAGFSTPFSFSNAQSTFTLGT<br>QAFSNQEVYIDRIEFVPAEVTF<br>EAESDLERAQKAVNALFTSTS<br>QLGLKTNVTGYHIDQVSNLVA<br>CLSDEFCLDEKRELSEKVKHA<br>KRLSDKRNLLQDPNFRGINRQ<br>PDHGWRGSTDITIQGGDDVFK<br>ENYVTLPGTFDECYPTYLYQKI<br>DESKLKAYTRYQLRGYIEDSQD<br>LEIYLIRYNSKHEIVNVPGTGSL<br>WPLSVENQIGPCGEPNRCAPH<br>LEWNPDLHCSCRDGEKCVHH<br>SHHFSLDIDVGCTDLNEDLGV<br>WLIFKIKTQDGHARLGNLEFL<br>EEEPLLGEALARVKRAEKKWR<br>DKREKLQLETNIVYKEAKESV<br>DALFVNSQYDRLQADTNIAMI<br>HAADKRVHRIREAYLPELSVIP<br>GVNAAIFEELEGRIFTAYSLYD<br>ARNVIKNGNFNNGLLCWNV<br>KGHVDVEEQNNHRSVLVIPE<br>WEAEVSQKVRVCPGRGYILRV<br>TAYKEGYGEGCVTIHEIEDNTD<br>ELKFSNCVEEGYPNNTVTCNE<br>YTMNQGVGECTDACNVRNR<br>GYEDAYGHNPSTPVHYTTPYE<br>EETYTDERRENPCEANKGYVN<br>YTPLPVGYVTKELEYFPETDTV<br>WIEIGETEGTFIVDSVELLLMEE

		tactatccaaggaggagatgacgtattcaaagaga<br>attacgttacgctaccgggtacttttgatgagtgctat<br>ccaacgtatttatatcaaaaaatagatgagtcgaaa<br>ttaaaagcctatacccgttatcaattaagagggtata<br>tcgaagatagtcaagacttagaaatctatttaattcg<br>ttacaattcaaaacacgaaatagtaaatgtaccagg<br>tacagggagtttatggcctctttctgtagaaaatcaa<br>attggaccttgtggagaaccgaatcgatgcgcgcc<br>acaccttgaatggaatcctgatttacactgttcctgca<br>gagacggggaaaaatgtgtgcatcattctcatcatt<br>tctctttggacattgatgtcggatgtacagatttaaat<br>gaggacctaggtgtatggttgatattcaagattaag<br>acgcaagatggccacgcaagactagggaatctag<br>agtttctcgaagaggaaccgttattaggcgaagcgt<br>tagcacgtgtgaagagagcggagaagaagtgga<br>gagacaaacgcgagaaactgcagttggaaacaaa<br>tattgtctataaagaggcaaaagaatctgtagatgc<br>tttatttgtaaactctcaatatgatagattacaagcgg<br>atacgaacatcgcgatgattcatgcggcagataaa<br>cgcgttcatagaatccgggaagcgtatctgccaga<br>gttgtctgtgattccaggtgtcaatgcggccatttcg<br>aagaattagagggacgtatttttacagcgtattcctt<br>atatgatgcgagaaatgttattaaaaatggcaatttc<br>aataatggcttattatgctggaacgtgaaagggcat<br>gtagatgtagaagagcaaaacaaccaccgttcggt<br>ccttgttatcccagaatgggaagcagaagtgtcaca<br>aaaagttcgtgtctgtccaggtcgtggttatatccttc<br>gtgttacagcgtacaaagagggatatggagaagg<br>ttgcgtaacgatccatgagatcgaagacaatacag<br>acgaattaaagtttagcaactgtgtagaagaagga<br>tatccaaacaacacggtaacgtgtaatgagtatact<br>atgaatcaaggggtaggagagtgtacggatgcat<br>gtaatgtccgtaatcgtggatatgaggatgcatatg<br>gacacaatccttcaacgcctgttcattacacaacgcc<br>gtacgaagaagaaacgtatacagatgaacgaaga<br>gagaatccttgtgaagctaacaaagggtatgtgaa<br>ttacacgccactaccagttggttatgtgacaaaaga<br>attagaatacttcccagaaaccgacacagtatggat<br>tgaaattggagaaacggaaggaacattcattgtgg<br>acagcgtggaattactccttatggaggaatag		
cry8Ea1	**3501**	atggagataagtgaccagaatcaatacatcccctat<br>aactgtttgaataatcctgaaagtgagatatttaatg<br>ctagaaattccaatttcggactggtttctcaagtcag<br>ctcgggacttacgcgttttcttctagaggcagctgtc<br>ccagaggctggttttgcacttggcctattcgatatcat<br>ttggggcgctctaggcgttgatcaatggagcctattt<br>cttaggcagattgagcaattaatacgacaagaaata<br>acagagttagaaaggaatagagcgactgcaatatt<br>aactggactatcgtcaagctataatctatatgttgag<br>gcgttaagagaatgggaaaatgatcctaataatcc<br>agcctcacaagaaagagtacgtacacgttttcgtct	**1166**	MEISDQNQYIPYNCLNNPESEI<br>FNARNSNFGLVSQVSSGLTRFL<br>LEAAVPEAGFALGLFDIIWGAL<br>GVDQWSLFLRQIEQLIRQEITE<br>LERNRATAILTGLSSSYNLYVE<br>ALREWENDPNNPASQERVRT<br>RFRLTDDAIVTGLPTLAIRNLE<br>VVNLSVYTQAANLHLSLLRDA<br>VYFGERWGLTQANIEDLYTRL<br>TSNIQEYSDHCARWYNQGLN<br>EIGGISRRYLDFQRDLTISVLDI<br>VALFPNYDIRTYPIPTQSQLTRE<br>IYTSPVVAGNINFGLSIANVLR<br>APHLMDFIDRIVIYTNSVRSTP

	aacggacgacgctatagtaacaggtttacctacttt<br>ggcaattcggaatcttgaggtagtgaatttatcagtc<br>tatactcaagcagcaaatctacacttatctttgttaag<br>agatgccgtttactttggagaaagatggggattaac<br>acaagcaaatattgaagatctgtacacaagactcac<br>gagtaatatccaagaatattcagaccattgtgcaag<br>atggtataatcaaggtttaaatgagattggagggat<br>aagtaggagatatttggacttccaaagagatttaac<br>aatttctgtcttagatattgtcgccctttcccaaattac<br>gatatccgaacatatcctattccgacacaaagtcaat<br>taacaagggagatttatacctctcccgtcgttgcag<br>gtaatataaattttggtttaagtatagcgaatgtattg<br>agagcccctcatctgatggactttattgatcgaatag<br>tcatttatacaaattcagttagaagtactccatattgg<br>gcagggcatgaagtcatatcgagaagaacaggg<br>caagggcaaggaaatgagataagatttcctttatat<br>ggagtggctgcaaatgcagaaccaccagttactat<br>aagacctacaggatttactgatgagcaacgacaat<br>ggtatagagcgcgatcgcgtgttgtctcgtttagaa<br>gttcaggtcaagactttagtttggtagatgccgtag<br>gattccttactatatttagcgctgtttcaatctatagaa<br>atggctttggatttaacactgatactattgatgaaatt<br>ccaattgagggaaccgatccattcactggatatagc<br>caccgattatgccatgtgggctttcttgcgtcatctcc<br>attcatcagtcagtatgcaagggctcctatatttctt<br>ggacgcaccgtagtgcaacccttacaaatacaattg<br>ctccagatgtcattacccaaataccgttagtaaagg<br>ctttcaatcttcattcaggtgccacgattgttaaagga<br>ccaggttttacaggtggggatatccttcgaagaacg<br>aatgttggtagctttggagatatgcgtgtaaacatta<br>ctgcaccactatcacaaagatatcgcgtaaggattc<br>gttatgcttctacgacagatttacaattctatacgaat<br>attaatggaactactattaatattggtaatttctcgag<br>cactatggacagtggggatgatttacagtacggaa<br>gattcagggttgcaggttttactactccatttaccttt<br>cagatgcaaacagcacattcacaataggtgcttttg<br>gcttctctccaaacaacgaagtttatatagatcgaat<br>tgaatttgtcccggcagaagtaacatttgaggcaga<br>atatgatttagagaaagctcagaaagcggtgaatg<br>cgctgtttacttcttccaatcaaatcgggttaaaaaca<br>gatgtgacggactatcatattgataaagtatccaatc<br>tagttgagtgtttatcagatgaattttgtctagatgaa<br>aagcgagaattgtccgagaaagtcaaacatgcga<br>agcgactcagtgatgagcggaatttacttcaagatc<br>caaacttcagaggcatcaatagacaaccagaccgt<br>ggttggagaggaagtacggatattaccatccaagg<br>aggagatgacgtattcaaagagaattacgttacgct<br>accgggtacctttgatgggtgctatccaacgtattta<br>tatcaaaaaatagatgagtcgaaattaaaagtctat<br>actcgttatcaattaagagggtatatcgaggatagt<br>caagacttagaaatctatttaattcgctacaatgcaa	YWAGHEVISRRTGQGQGNEIR<br>FPLYGVAANAEPPVTIRPTGFT<br>DEQRQWYRARSRVVSFRSSGQ<br>DFSLVDAVGFLTIFSAVSIYRNG<br>FGFNTDTIDEIPIEGTDPFTGYS<br>HRLCHVGFLASSPFISQYARAP<br>IFSWTHRSATLTNTIAPDVITQI<br>PLVKAFNLHSGATIVKGPGFT<br>GGDILRRTNVGSFGDMRVNIT<br>APLSQRYRVRIRYASTTDLQFY<br>TNINGTTINIGNFSSTMDSGDD<br>LQYGRFRVAGFTTPFTFSDANS<br>TFTIGAFGFSPNNEVYIDRIEFV<br>PAEVTFEAEYDLEKAQKAVNA<br>LFTSSNQIGLKTDVTDYHIDKV<br>SNLVECLSDEFCLDEKRELSEK<br>VKHAKRLSDERNLLQDPNFR<br>GINRQPDRGWRGSTDITIQGG<br>DDVFKENYVTLPGTFDGCYPT<br>YLYQKIDESKLKVYTRYQLRGY<br>IEDSQDLEIYLIRYNAKHETVN<br>VPGTGSLWPLSAQSPIGKCGEP<br>NRCAPHLEWNPDLDCSCRNG<br>EKCAHHSHHFSLDIDVGCTDL<br>NEDLGVWVIFKIKTQDGHARL<br>GNLEFLEEKPLLGEALARVKR<br>AEKKWRDKREKLELETNIVYK<br>EAKESVDALFVNSQYDQLQA<br>DTNIAMIHAADKRVHSIREAY<br>LPELSVIPGVNAAIFEELEGRIF<br>TAFSLYDARNVIKNGDFNNGL<br>SCWNVKGHVDVEEQNNHRS<br>VLVVPEWEAEVSQEVRVCPGR<br>GYILRVTAYKEGYGEGCVTIHE<br>IENNTDELKFSNCVEEEVYPN<br>NTVTCNDYTANQEEYKGAYTS<br>HNRGYDEAYGNNPSVPADYT<br>PVYEEKAYTDGRRENPCESNR<br>GYGDYTPLPAGYVTKELEYFPE<br>TDKVWIEIGETEGTFIVESVELL<br>LMEE

		aacacgaaacagtaaatgtgccaggtacgggttcc<br>ttatggccgctttcagcccaaagtccaatcggaaag<br>tgtggagagccgaatcgatgcgcgccacaccttga<br>atggaatcctgacttagattgttcgtgtaggaatgg<br>agaaaagtgtgcccatcattcgcatcatttctccttag<br>acattgatgttggatgtacagatctaaatgaggacc<br>taggtgtatgggtgatctttaagattaagacgcaag<br>atggtcatgcaagattaggaaatctagagtttctcg<br>aagagaaaccattattaggggaagcactagctcgt<br>gtgaaaagagcggagaaaaaatggagagacaa<br>acgtgaaaaattggaattggaaacaaatattgtttat<br>aaagaggcaaaagaatctgtagatgctttatttgta<br>aactctcaatatgatcaattacaagcggatacgaat<br>atcgcgatgattcatgcggcagataaacgcgttcat<br>agcattcgagaagcgtatctgccggagctgtctgtg<br>attccgggtgtcaatgcggcgattttgaagaattag<br>aagggcgtattttcactgcattctccctatatgatgcg<br>agaaatgtcattaaaaatggcgatttcaataatggc<br>ttatcatgctggaacgtgaaagggcatgtagatgta<br>gaagaacagaacaaccatcgttcggtccttgttgttc<br>cagaatgggaagcagaagtgtcacaagaagttcg<br>tgtttgtccgggtcgtggttatatccttcgtgtcacag<br>cgtacaaggagggatatggagaaggttgcgtaac<br>cattcatgagatcgagaacaatacagacgaactga<br>aattcagcaactgcgtagaagaggaagtatatcca<br>aacaacacggtaacgtgtaatgattatactgcaaat<br>caagaagaatacaaaggtgcgtacacttctcataat<br>cgaggatatgacgaagcctatggaaataacccttc<br>cgtaccagcagattatacgccagtctatgaagaaa<br>aagcgtatacagatggacgaagagagaatccttgt<br>gaatctaacagagggtatggggattacacgccact<br>accagctggttatgtgacgaaggaattagagtactt<br>cccagaaaccgataaggtatggattgagattggag<br>aaacggaaggaacatttatcgtggagagcgtgga<br>attactccttatggaggaatag		
cry1Ib	**2160**	atgaaactaaagaatccagataagcatcaaagctt<br>gtctagcaatgcgaaagtagataaaatcgctacgg<br>attcactaaaaaatgaaacagatatagaattgaaaa<br>atatgaataatgaagattatttgagaatgtctgagc<br>atgagagtattgatccgtttgttagtgcatcaacaatt<br>caaacgggtattggaattgctggtaagattcttggt<br>actctaggtgttccttttgctggacaaatagctagcct<br>ctatagttttatcttaggcgagctttggcctaaaggg<br>aaaagtcaatgggaaatctttatggaacatgtaga<br>agagattattaatcaaaaaatattaacttatgcaaga<br>aataaagcactttcagacttgagaggattaggtgat<br>gctttagccgtctaccatgaatcgcttgaaagttggg<br>ttgaaaatcgtaataacactcgagcgaggagtgta<br>gtcaagaaccaatatatcgcattagaactgatgtttg<br>ttcaaaaactaccttcttttgcagtatctggtgaggaa<br>gtaccattattaccgatatatgcccaagctgccaattt	**719**	MKLKNPDKHQSLSSNAKVDK<br>IATDSLKNETDIELKNMNNED<br>YLRMSEHESIDPFVSASTIQTGI<br>GIAGKILGTLGVPFAGQIASLYS<br>FILGELWPKGKSQWEIFMEHV<br>EEIINQKILTYARNKALSDLRG<br>LGDALAVYHESLESWVENRN<br>NTRARSVVKNQYIALELMFVQ<br>KLPSFAVSGEEVPLLPIYAQAA<br>NLHLLLLRDASIFGKEWGLSA<br>SEISTFYNRQVERTRDYSDHCI<br>KWYNTGLNNLRGTNAKSWV<br>RYNQFRKDMTLMVLDLVALFP<br>SYDTLVYPIKTTSQLTREVYTD<br>AIGTVHPNQAFASTTWYNNN<br>APSFSAIEAAVIRSPHLLDFLEK<br>VTIYSLLSRWSNTQYMNMWG<br>GHRLESRPIGGALNTSTQGST<br>NTSINPVTLQFTSRDVYRTESW<br>AGLNLFLTQPVNGVPRVDFH

		acatttgttgttattaagagatgcatctattttggaaaagaatggggattatcagcttcagaaatttcaacattttataaccgtcaagtcgaacgaacaagagattattccgaccattgtataaaatggtataatacaggcctaaataacttgaggggtacaaatgccaaaagttgggttcgttataatcaatttcgtaaagatatgacattaatggtattagatttagttgcgctattcccaagctatgatacacttgtatatcctattaaaaccacttcacaacttacaagagaagtatatacagacgcaattgggaccgtgcatccgaatcaagcttttgcaagtacgacttggtataataataatgcaccttcgttctctgccatagaggctgctgttatccgaagtccacacctacttgatttctagaaaaagttacaatttacagcttattaagtcggtggagtaatactcagtatatgaatatgtggggaggacatagacttgaatcccgcccaataggaggggcattaaatacctcaacacaaggatctaccaatacttcgattaatccagtaacattacagttcacgtctcgagacgtttataggactgaatcatgggcagggctgaatttattttaactcaacctgttaatggagtacctagagttgatttccattggaaatttcccacgctaccaatagcatctgataattttattatctagggtatgctggagttggtacgcaattacaagattcagaaaatgaattaccacctgaaacaacaggacagccaaattatgaatcatatagtcatagattatcccatataggactcatttcagcatcccacgtgaaagcattggtatattcttggacacatcgtagtgcagatcgtacaaatacaattgagccaaatagcattacacaaataccattagtaaaagcgttcaatctgtcttcaggtgccgctgtagtgagaggaccaggatttacaggtggggatatccttcgaagaacgaatactggtacatttggggatatacgagtaaatattaatccaccatttgcacaaagatatcgcgtgaggattcgctatgcttctactacagatttacaattccatacgtcaattaacggtaaagctattaatcaaggtaatttttcagcaactatgaatagaggagaggacttagactataaaacctttagaactgtaggctttaccactccatttagcttttcagatgtacaaagtacattcacaataggtgcttggaacttctcttcaggtaacgaagtttatatagatcgaattgaatttgttccggtagaagtaacatatgaggcagaatatgattttgaaaaagcgcaagagaaggttactgcactgtttacatctacgaatccaagaggattaaaaacagatgtaaaggattatcatattgaccaggtatcaaatttagtagagtctctatcagatgaattctatcttgatgaaaagagagaattattcgagatagttaaatacgcgaagcaaatccatattgagcgtaacatgtag		WKFPTLPIASDNFYYLGYAGVGTQLQDSENELPPETTGQPNYESYSHRLSHIGLISASHVKALVYSWTHRSADRTNTIEPNSITQIPLVKAFNLSSGAAVVRGPGFTGGDILRRTNTGTFGDIRVNINPPFAQRYRVRIRYASTTDLQFHTSINGKAINQGNFSATMNRGEDLDYKTFRTVGFTTPFSFSDVQSTFTIGAWNFSSGNEVYIDRIEFVPVEVTYEAEYDFEKAQEKVTALFTSTNPRGLKTDVKDYHIDQVSNLVESLSDEFYLDEKRELFEIVKYAKQIHIERNM
cry2Ab	**1902**	atgaatagtgtattgaatagcggaagaactactatttgtgatgcgtataatgtagcggctcatgatccatttagttttcaacacaaatcattagataccgtacaaaaggaatggacggagtggaaaaaaaataatcatagtttatacctagatcctattgttggaactgtggctagttttctgttaaagaaagtggggagtcttgttggaaaaaggatactaagtgagttacggaatttaatatttcctagtggt	**633**	MNSVLNSGRTTICDAYNVAAHDPFSFQHKSLDTVQKEWTEWKKNNHSLYLDPIVGTVASFLLKKVGSLVGKRILSELRNLIFPSGSTNLMQDILRETEKFLNQRLNTDTVARVNAELTGLQANVEEFNRQVDNFLNPNRNAVPLSITSSVNTMQQLFLNRLPQFQMQGYQLLLLPLFAQAANLHLSFI

		agtacaaatctaatgcaagatatttaagagagaca<br>gaaaaattcctgaatcaaagacttaatacagacact<br>gttgcccgtgtaaatgcggaattgacagggctgca<br>agcaaatgtagaagagtttaatcgacaagtagata<br>atttttgaaccctaaccgaaacgctgttcctttatcaa<br>taacttcttcagttaatacaatgcaacaattatttctaa<br>atagattaccccagttccagatgcaaggataccaac<br>tgttattattacctttatttgcacaggcagccaatttac<br>atctttcttttattagagatgttattctaaatgcagatg<br>aatggggaatttcagcagcaacattacgtacgtatc<br>gagattacttgaaaaattatacaagagattactctaa<br>ctattgtataaatacgtatcaaagtgcgtttaaaggt<br>ttaaacactcgtttacacgatatgttagaatttagaac<br>atatatgtttttaaatgtatttgaatatgtatctatctg<br>gtcgttgtttaaatatcaaagtcttctagtatcttccgg<br>tgctaatttatatgcaagtggtagtggaccacagca<br>gacccaatcatttacttcacaagactggccattttat<br>attctcttttccaagttaattcaaattatgtgttaaatg<br>gatttagtggtgctaggctttctaataccttccctaat<br>atagttggtttacctggttctactacaactcacgcatt<br>gcttgctgcaagggttaattacagtggaggaatttc<br>gtctggtgatataggtgcatctccgtttaatcaaaatt<br>ttaattgtagcacatttctccccccattgttaacgccat<br>ttgttaggagttggctagattcaggttcagatcggg<br>agggcgttgccaccgttacaaattggcaaacagaa<br>tcctttgagacaactttagggttaaggagtggtgctt<br>ttacagctcgcggtatttcaaactatttcccagattatt<br>ttattcgtaatatttctggagttcctttagttgttagaa<br>atgaagatttaagaagaccgttacactataatgaaa<br>taagaaatatagcaagtccttcaggaacacctggtg<br>gagcacgagcttatatggtatctgtgcataacagaa<br>aaaataatatccatgccgttcatgaaaatggttctat<br>gattcatttagcgccaaatgactatacaggatttact<br>atttcgccgatacatgcaactcaagtgaataatcaa<br>acacgaacatttatttctgaaaaatttggaaatcaag<br>gtgattccttaaggtttgaacaaaataacacgacag<br>ctcgttatacgcttagagggaatggaaatagttaca<br>atctttatttaagagtttcttcaataggaaattccacta<br>ttcgagttactataaacggtagggtatatactgctac<br>aaatgttaatactactacaaataacgatggagttaat<br>gataacggagctcgtttttcagatattaatatcggta<br>atgtagtagcaagtagtaattctgatgtaccattaga<br>tataaatgtaacattaaactccggtactcaatttgatc<br>ttatgaatattatgcttgtaccaactaatatttcaccac<br>tttattaa		RDVILNADEWGISAATLRTYR<br>DYLKNYTRDYSNYCINTYQSA<br>FKGLNTRLHDMLEFRTYMFLN<br>VFEYVSIWSLFKYQSLLVSSGA<br>NLYASGSGPQQTQSFTSQDWP<br>FLYSLFQVNSNYVLNGFSGAR<br>LSNTFPNIVGLPGSTTTHALLA<br>ARVNYSGGISSGDIGASPFNQN<br>FNCSTFLPPLLTPFVRSWLDSG<br>SDREGVATVTNWQTESFETTL<br>GLRSGAFTARGISNYFPDYFIR<br>NISGVPLVVRNEDLRRPLHYN<br>EIRNIASPSGTPGGARAYMVSV<br>HNRKNNIHAVHENGSMIHLA<br>PNDYTGFTISPIHATQVNNQTR<br>TFISEKFGNQGDSLRFEQNNTT<br>ARYTLRGNGNSYNLYLRVSSIG<br>NSTIRVTINGRVYTATNVNTTT<br>NNDGVNDNGARFSDINIGNV<br>VASSNSDVPLDINVTLNSGTQF<br>DLMNIMLVPTNISPLY
cry9Ba	**3354**	atgtccgaaggagactatatagactcgtatataaat<br>cctggaaatgttagaactggactacaaactggaatt<br>gatattgttgcagtagtagtaggtgctttaggtgga<br>ccagttggtggcatactcactggttttctttctactcttt<br>ttggttttctttggccatctaatgatcaagcagtatgg<br>gaagcttttatagaacaaatggaagaactgattgaa	**1117**	MSEGDYIDSYINPGNVRTGLQ<br>TGIDIVAVVVGALGGPVGGILT<br>GFLSTLFGFLWPSNDQAVWEA<br>FIEQMEELIEQRISDQVVRTAL<br>DDLTGIQNYYNQYLIALKEWE<br>ERPNGVRANLVLQRFEILHAL<br>FVSSMPSFGSGPGSQRFQAQLL<br>VVYAQAANLHLLLLADAEKY

	caaaggatatcagatcaagtagtaaggactgcact<br>cgatgacttaactggaattcaaaattattataatcaat<br>atctaatagcattaaaggaatgggaggaaagacc<br>aaacggcgtaagagcaaacttagttttgcaaagatt<br>tgaaatcttgcacgcgctatttgtaagtagtatgcca<br>agttttggtagtggccctggaagtcaaaggtttcag<br>gcacaattgttggttgtttatgcgcaagcagcaaatc<br>ttcatttactattattagctgatgctgaaaagtatggg<br>gcaagatggggactccgtgaatcccagatagaatt<br>atattttaatgaactacaaactcgtactcgagattac<br>accaaccattgtgtaaacgcgtataataacgggtta<br>gccgggttacgaggaacgagcgctgaaagttggt<br>taaagtaccatcaattccgcagagaagcaaccttaa<br>tggcaatggatttgatagctttatttccatattataac<br>acccggcgatatccaatcgcagtaaatcctcagctt<br>acacgtgaggtatatacagatccattaggcgttcctt<br>ctgaagaatcaagtttatttccagaattgagatgctt<br>aagatggcaagagacttctgccatgactttttcaaat<br>ttggaaaatgcaataattcgtccaccacatctatttg<br>acacaataaacaatttaatgatttataccggttcttttc<br>gcttcactaacaatcaattaattgaagggtggattg<br>gacattctgtaactagtagtttgttggccagtggacc<br>aacaacagtagtgagaagaaattacggtagcacg<br>acatctattgtaaactatttagttttaatgatcgtgat<br>gtttatcagattaatacgagatcacatactgggttgg<br>gattccagaacgcacctttatttggaatcactagagc<br>tcaattttacccaggtgggacttattcagtaactcaac<br>gaaatgcattaacatgtgaacaaaattataattcaat<br>tgatgagttaccgagcctagacccaaatgaacctat<br>cagtagaagttatagtcatagattatctcatattacct<br>cctatttgcatcgtgtattgactattgatggtattaata<br>tatattcaggaaatctccctacttatgtatggacccat<br>cgcgatgtggaccttacaaacacgattaccgcagat<br>agaattacacaactaccattggtaaagtcatttgaaa<br>tacctgcgggtactactgtcgtaagaggaccaggtt<br>ttacaggaggggatatactccgaagaacaggggtt<br>ggtacatttggaacaataagggtaaggactactgc<br>ccccttaacacaaagatatcgcataagattccgtttc<br>gcttctaccacaaatttgttcattggtataagagttgg<br>tgatagacaagtaaattattttgacttcggaagaaca<br>atgaacagaggagatgaattaaggtacgaatctttt<br>gctacaagggagtttactactgattttaattttagaca<br>acctcaagaattaatctcagtgtttgcaaatgcattta<br>gcgctggtcaagaagtttattttgatagaattgagat<br>tatccccgttaatcccgcacgagaggcgaaagagg<br>atctagaagcagcaaagaaagcggtggcgagctt<br>gtttacacgcacaagggacggattacaagtaaatg<br>tgaaagattatcaagtcgatcaagcggcaaatttag<br>tgtcatgcttatcagatgaacaatatgggtatgaca<br>aaaagatgttattggaagcggtacgcgcggcaaa<br>acgcctcagccgagaacgtaacttacttcaggatcc	GARWGLRESQIELYFNELQTRT<br>RDYTNHCVNAYNNGLAGLRG<br>TSAESWLKYHQFRREATLMA<br>MDLIALFPYYNTRRYPIAVNPQ<br>LTREVYTDPLGVPSEESSLFPEL<br>RCLRWQETSAMTFSNLENAIIR<br>PPHLFDTINNLMIYTGSFRFTN<br>NQLIEGWIGHSVTSSLLASGPT<br>TVVRRNYGSTTSIVNYFSFNDR<br>DVYQINTRSHTGLGFQNAPLF<br>GITRAQFYPGGTYSVTQRNAL<br>TCEQNYNSIDELPSLDPNEPISR<br>SYSHRLSHITSYLHRVLTIDGIN<br>IYSGNLPTYVWTHRDVDLTNT<br>ITADRITQLPLVKSFEIPAGTTV<br>VRGPGFTGGDILRRTGVGTFG<br>TIRVRTTAPLTQRYRIRFRFAST<br>TNLFIGIRVGDRQVNYFDFGRT<br>MNRGDELRYESFATREFTTDF<br>NFRQPQELISVFANAFSAGQE<br>VYFDRIEIIPVNPAREAKEDLE<br>AAKKAVASLFTRTRDGLQVNV<br>KDYQVDQAANLVSCLSDEQY<br>GYDKKMLLEAVRAAKRLSRER<br>NLLQDPDFNTINSTEENGWKA<br>SNGVTISEGGPFYKGRALQLAS<br>ARENYPTYIYQKVDASELKPYT<br>RYRLDGFVKSSQDLEIDLIHHH<br>KVHLVKNVPDNLVSDTYPDDS<br>CSGINRCQEQQMVNAQLETE<br>HHHPMDCCEAAQTHEFSSYI<br>DTGDLNSSVDQGIWAIFKVRT<br>TDGYATLGNLELVEVGPLSGES<br>LEREQRDNTKWSAELGRKRA<br>ETDRVYQDAKQSINHLFVDYQ<br>DQQLNPEIGMADIMDAQNLV<br>ASISDVYSDAVLQIPGINYEIYT<br>ELSNRLQQASYLYTSRNAVQN<br>GDFNNGLDSWNATAGASVQ<br>QDGNTHFLVLSHWDAQVSQQ<br>FRVQPNCKYVLRVTAEKVGGG<br>DGYVTIRDGAHHTETLTFNAC<br>DYDINGTYVTDNTYLTKEVIFY<br>SHTEHMWVEVNETEGAFHID<br>SIEFVETEK

		agattttaatacaatcaatagtacagaagaaaatgg<br>atggaaagcaagtaacggcgttactattagtgagg<br>gcggtccattctataaaggccgtgcacttcagctag<br>caagtgcacgagaaaattatccaacatacatttatc<br>aaaaagtagatgcatcggagttaaaaccttatacac<br>gatatagactagatgggttcgtgaagagtagtcaa<br>gatttagaaattgatctcattcaccatcataaagtcca<br>tcttgtgaaaaatgtaccagataatttagtatctgata<br>cttacccagatgattcttgtagtggaatcaatcgatg<br>tcaggaacaacagatggtaaatgcgcaactggaa<br>acagagcatcatcatccgatggattgctgtgaagca<br>gctcaaacacatgagttttcttcctatattgatacagg<br>ggatttaaattcgagtgtagaccagggaatctggg<br>cgatctttaaagttcgaacaaccgatggttatgcga<br>cgttaggaaatcttgaattggtagaggtcggaccgt<br>tatcgggtgaatctttagaacgtgaacaaagggat<br>aatacaaaatggagtgcagagctaggaagaaag<br>cgtgcagaaacagatcgcgtgtatcaagatgccaa<br>acaatccatcaatcatttatttgtggattatcaagatc<br>aacaattaaatccagaaatagggatggcagatatt<br>atggacgctcaaaatcttgtcgcatcaatttcagatg<br>tatatagcgatgccgtactgcaaatccctggaatta<br>actatgagatttacacagagctgtccaatcgcttaca<br>acaagcatcgtatctgtatacgtctcgaaatgcggt<br>gcaaaatggggactttaacaacgggctagatagct<br>ggaatgcaacagcgggtgcatcggtacaacagga<br>tggcaatacgcatttcttagttctttctcattgggatgc<br>acaagtttctcaacaatttagagtgcagccgaattgt<br>aaatatgtattacgtgtaacagcagagaaagtagg<br>cggcggagacggatacgtgactatccgggatggt<br>gctcatcatacagaaacgcttacatttaatgcatgtg<br>attatgatataaatggcacgtacgtgactgataata<br>cgtatctaacaaaagaagtgatattctattcacatac<br>agaacacatgtgggtagaggtaaatgaaacagaa<br>ggtgcatttcatatagatagtattgaattcgttgaaa<br>cagaaaagtaa		
Partial sequences				
**cry9Aa**	**276**	gtgcagccgaattgtaaatatgtattacgtgtaaca<br>gcagagaaagtaggcggcggagacggatacgtg<br>actatccgggatgatgctcatcatacagaaacgctt<br>acatttaatgcatgtgattatgatataaatggcacgt<br>acgtgactgataatacgtatctaacaaaagaagtg<br>gtattccatccggagacacaacacatgtgggtaga<br>ggtaaatgaaacagaaggtgcatttcatatagata<br>gtattgaattcgttgaaacagaaaagtaa	**91**	MQPNCKYVLRVTAEKVGGGD<br>GYVTIRDDAHHTETLTFNACD<br>YDINGTYVTDNTYLTKEVVFH<br>PETQHMWVEVNETEGAFHID<br>SIEFVETEK
**cry1Ac**	**135**	atggagagaccaacacgaaaaattacatttgtaaa<br>ctcccgatatgatagtttacaaatggatacgaacatt<br>gcaatgatttatacggccgataaacgcgttcataga<br>aaccgagaagcgtatctttcagagtga	**44**	MERPTRKITFVNSRYDSLQMD<br>TNIAMIYTADKRVHRNREAYL<br>SE
